# Smoking-attributable burden of musculoskeletal disorders, 1990–2021, with exploratory projections to 2050: A secondary dataset analysis of the Global Burden of Disease Study 2021

**DOI:** 10.18332/tid/226288

**Published:** 2026-08-25

**Authors:** Zhihong Fu, Yunhui Zhang, Zhaojun Chen

**Affiliations:** 1 Third Affiliated Hospital of Beijing University of Chinese Medicine, Beijing, China; 2 Beijing University of Chinese Medicine, Beijing, China; 3 Engineering Research Center of Intelligent Orthopedics and Sports Rehabilitation, Ministry of Education, Beijing, China

**Keywords:** smoking, musculoskeletal disorders, disability-adjusted life years, socioeconomic inequality, Global Burden of Disease

## Abstract

**INTRODUCTION:**

Musculoskeletal disorders (MSDs) are a leading source of disability worldwide, but the population-level burden attributable to smoking remains insufficiently characterized. The aim of this study was to quantify global, regional, and national smoking-attributable burden of MSDs from 1990 to 2021, and to explore socioeconomic inequalities and projections to 2050.

**METHODS:**

This secondary dataset analysis used Global Burden of Disease Study 2021 estimates for smoking-attributable DALYs due to MSDs. Eligible estimates included both sexes combined, the risk factor smoking, the aggregate cause category of MSDs, all-age DALYs, age-standardized DALYs rate (ASDR), age-specific rates, global, sociodemographic index (SDI), regional, and national locations, for years 1990–2021. Temporal trends were summarized using estimated annual percentage change (EAPC) and joinpoint regression. Socioeconomic inequality, frontier performance, and exploratory autoregressive integrated moving average projections to 2050 were assessed; Bayesian age-period-cohort forecasts were used as sensitivity analysis for global DALYs.

**RESULTS:**

Global smoking-attributable DALYs due to MSDs increased from 6.82 million (95% UI: 3.85–10.12) in 1990 to 8.89 million (95% UI: 4.72–13.68) in 2021, whereas ASDR decreased from 153.57 (95% UI: 85.94–228.28) to 102.44 (95% UI: 54.60–157.30). The global EAPC in ASDR was -1.20% (95% CI: -1.24 – -1.16). The largest relative increases in DALYs occurred in Middle-SDI regions (89.15%) and Low-SDI regions (71.71%). High-SDI regions retained the highest ASDR in 2021 (154.13; 95% UI: 81.19–231.32). Exploratory projections suggested that global ASDR may decline to 45.40 (95% UI: 29.06–61.74) by 2050, while DALYs may reach 7.53 million (95% UI: 4.12–10.93).

**CONCLUSIONS:**

Smoking-attributable MSDs remain a measurable component of global disability burden. The persistence of absolute burden despite declining ASDR may warrant greater consideration in future tobacco-related burden assessments and rehabilitation planning, while causal and policy interpretations warrant cautious interpretation as this is an ecological modelling study.

## INTRODUCTION

Musculoskeletal disorders are a major contributor to global non-fatal health loss^1^. They generate substantial rehabilitation needs across countries with different levels of development^2^. Because musculoskeletal conditions compromise mobility, independence, and work participation, they have become an important challenge for healthy ageing^3^. Their economic consequences are also considerable in national health systems^4^. At the population level, MSDs collectively represent a sustained public health burden across diverse settings^5^.

Smoking is a biologically plausible contributor to several musculoskeletal outcomes through pathways involving systemic inflammation, impaired bone metabolism, reduced tissue perfusion, and delayed repair^6^. Earlier epidemiological work linked smoking with low back pain^7^. Experimental and translational evidence also suggests that nicotine can affect bone cells and fracture repair^8^. Smoking has been associated with a higher risk of rheumatoid arthritis^9^. Meta-analytic evidence further supports an association between smoking and low back pain^10^. Smoking-related skeletal vulnerability also includes increased fracture risk^11^.

Although the Global Burden of Disease framework provides standardized estimates of disease burden attributable to major risks, smoking-attributable musculoskeletal disability has been less prominently described than other tobacco-induced disease categories^12^. Recent burden analyses for osteoarthritis highlight the need for forward-looking assessment of musculoskeletal disability^13^. The aim of this secondary dataset analysis was to quantify the global, regional, and national burden of MSDs attributable to smoking from 1990 to 2021, characterize temporal and socioeconomic patterns, and provide cautious exploratory projections to 2050 using GBD 2021 estimates.

## METHODS

### Study design and data source

This was a secondary dataset analysis of publicly available estimates from the Global Burden of Disease Study 2021 comparative risk assessment framework^14^. The study used GBD-provided smoking-attributable DALYs estimates and therefore represents population-level attributable burden within the GBD framework rather than individual-level causal effects.

Eligible estimates were extracted for the aggregate cause category of MSDs, the risk factor smoking, both sexes combined, years 1990–2021, and global, SDI quintile, GBD regional, and national locations. Outcomes included all-age DALYs, age-standardized DALYs rate per 100000 population (ASDR), and age-specific DALYs rate where applicable. MSDs were analyzed at the aggregate GBD cause level, consistent with recent GBD 2021 reporting on MSDs^15^.

Because the study used publicly available, de-identified secondary estimates, ethical approval and informed consent were not required.

### GBD case definition and smoking attribution

In GBD 2021, musculoskeletal disorders are represented as an aggregate cause category comprising disabling non-fatal conditions, including low-back pain, neck pain, osteoarthritis, rheumatoid arthritis, gout, and other MSDs. The present analysis used the GBD cause category of MSDs and did not reclassify individual disease subtypes^15^.

Within the GBD comparative risk assessment framework, smoking-attributable burden is estimated using population attributable fractions derived from the exposure distribution for smoking, the theoretical minimum risk exposure level, and relative risk estimates linking smoking to specific outcomes. The theoretical minimum risk exposure level for smoking is no exposure to smoked tobacco. Therefore, the DALYs and ASDR analyzed in this study represent GBD-estimated smoking-attributable burden of MSDs, rather than estimates recalculated from individual-level smoking data^14^.

### Statistical analysis

The primary outcomes were all-age DALYs and ASDR. Estimates are presented with 95% uncertainty intervals (UIs). Temporal trends in ASDR were first quantified using EAPC. A log-linear regression model was fitted to the annual ASDR series as: ln (ASDR_t_) = α + βt + ε_t_, where ASDR_t_ denotes the ASDR in year t, β is the regression coefficient for calendar year, and ε_t_ is the random error term.

The EAPC was calculated as EAPC=100×[exp(β) - 1], and its 95% confidence interval (CI) was calculated as 95% CI=100×[exp(β ± 1.96 × SE_β_) - 1], where SE_β_ is the standard error of β. An EAPC with 95% CI >0 was interpreted as an increasing trend, whereas an EAPC with 95% CI <0 was interpreted as a decreasing trend^16^.

Joinpoint regression was additionally used to identify temporal segments in the global and SDI-specific ASDR series. Annual percentage change (APC) was estimated for each segment, and average annual percentage change (AAPC) summarized the overall trend across 1990–2021. APCs, AAPCs, 95% CIs, and corresponding p values are reported.

To examine socioeconomic inequality, countries were ranked by SDI and inequality was summarized using regression-based measures conceptually aligned with slope-index and concentration-curve approaches^17^. Frontier analysis compared each country or territory with the estimated lowest attainable ASDR at a comparable SDI level; the effective difference from the frontier was interpreted as potential room for improvement rather than a causal health-system performance measure.

Exploratory time-series projections were generated using autoregressive integrated moving average (ARIMA) models^18^. Bayesian age-period-cohort (BAPC) forecasts were used only as a sensitivity analysis for global DALYs because the available BAPC rate series did not share the same baseline as the GBD ASDR series. Long-horizon smoking projections were interpreted cautiously in line with recent GBD forecasting work^19^. General population and risk-factor projection methods from GBD studies also informed the cautious interpretation of long-term forecasts^20^.

Statistical analyses and data visualization were performed using R software (version 4.3.1) and Python (version 3.11.9). ARIMA modeling was performed using the R forecast package (version 8.22.0), Bayesian age-period-cohort sensitivity analysis was performed using the R BAPC package, and joinpoint regression was performed using the Joinpoint Regression Program (version 5.1.0.0). A two-sided p<0.05 was considered statistically significant.

## RESULTS

### Global burden

**Figure 1:**
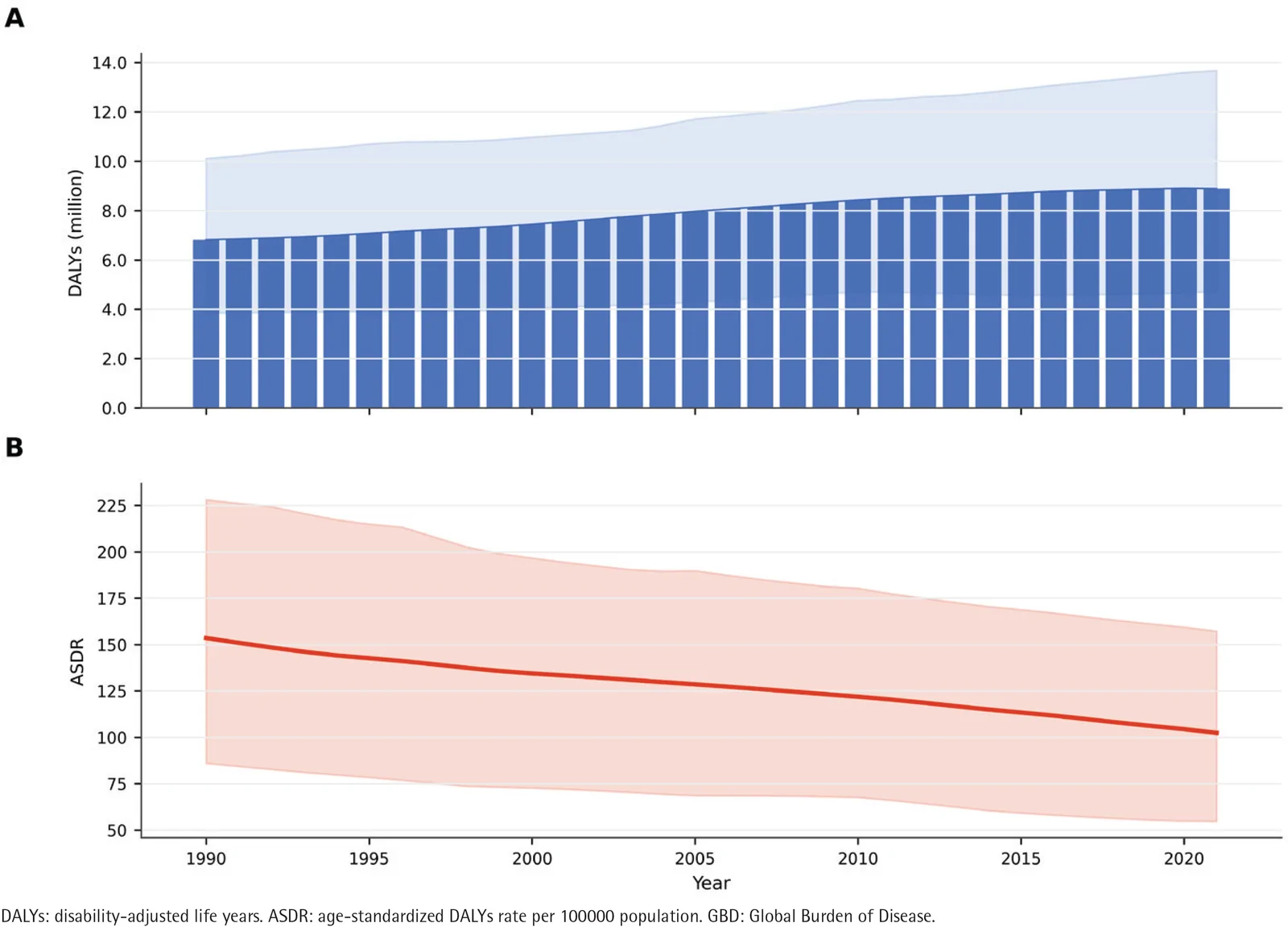
Global trends in DALYs and ASDR for smoking-attributable musculoskeletal disorders in a secondary dataset analysis of GBD 2021 estimates, 1990–2021: A) global DALYs with 95% uncertainty intervals; B) global ASDR with 95% uncertainty intervals

Globally, smoking-attributable DALYs due to MSDs increased from 6.82 million (95% UI: 3.85–10.12) in 1990 to 8.89 million (95% UI: 4.72–13.68) in 2021 (Table 1 and Figure 1). During the same period, ASDR decreased from 153.57 (95% UI: 85.94–228.28) to 102.44 (95% UI: 54.60–157.30), with a global EAPC of -1.20% (95% CI: -1.24 – -1.16) (Table 1 and Figure 1).

**Table 1:** Global and SDI-stratified burden of smoking-attributable musculoskeletal disorders in a secondary dataset analysis of GBD 2021 estimates, 1990 and 2021

*Location*	*1990* *DALYs (million)* *(95% UI)*	*2021* *DALYs (million)* *(95% UI)*	*Percent* *change*	*1990* *ASDR (95% UI)*	*2021* *ASDR (95% UI)*	*EAPC (95% CI)*
**Global SDI region**	6.82 (3.85–10.12)	8.89 (4.72–13.68)	30.33	153.57 (85.94–228.28)	102.44 (54.60–157.30)	-1.20 (-1.24 – -1.16)
**SDI region**						
High	4.56 (2.60–6.73)	5.45 (2.82–8.31)	19.45	209.92 (120.23–309.62)	154.13 (81.19–231.32)	-0.88 (-0.91 – -0.85)
High-middle	1.01 (0.58–1.52)	1.39 (0.77–2.12)	37.46	116.65 (67.25–178.02)	70.86 (39.25–108.41)	-1.49 (-1.52 – -1.47)
Middle	0.43 (0.24–0.64)	0.81 (0.44–1.22)	89.15	104.78 (59.10–157.65)	84.43 (46.03–128.32)	-0.64 (-0.70 – -0.57)
Low-middle	0.42 (0.22–0.65)	0.56 (0.30–0.92)	33.51	88.11 (46.03–136.61)	55.58 (29.64–91.59)	-1.37 (-1.48 – -1.25)
Low	0.39 (0.21–0.61)	0.67 (0.37–1.07)	71.71	85.32 (44.87–131.92)	63.16 (34.35–101.01)	-0.94 (-1.03 – -0.85)

DALYs: disability-adjusted life years; ASDR: age-standardized DALYS rate per 100000 population. SDI: sociodemographic index. GBD: Global Burden of Disease. UI: uncertainty interval. CI: confidence interval. EAPC: estimated annual percentage change. Values are for both sexes combined.

### Geographical distribution across countries and territories

**Figure 2:**
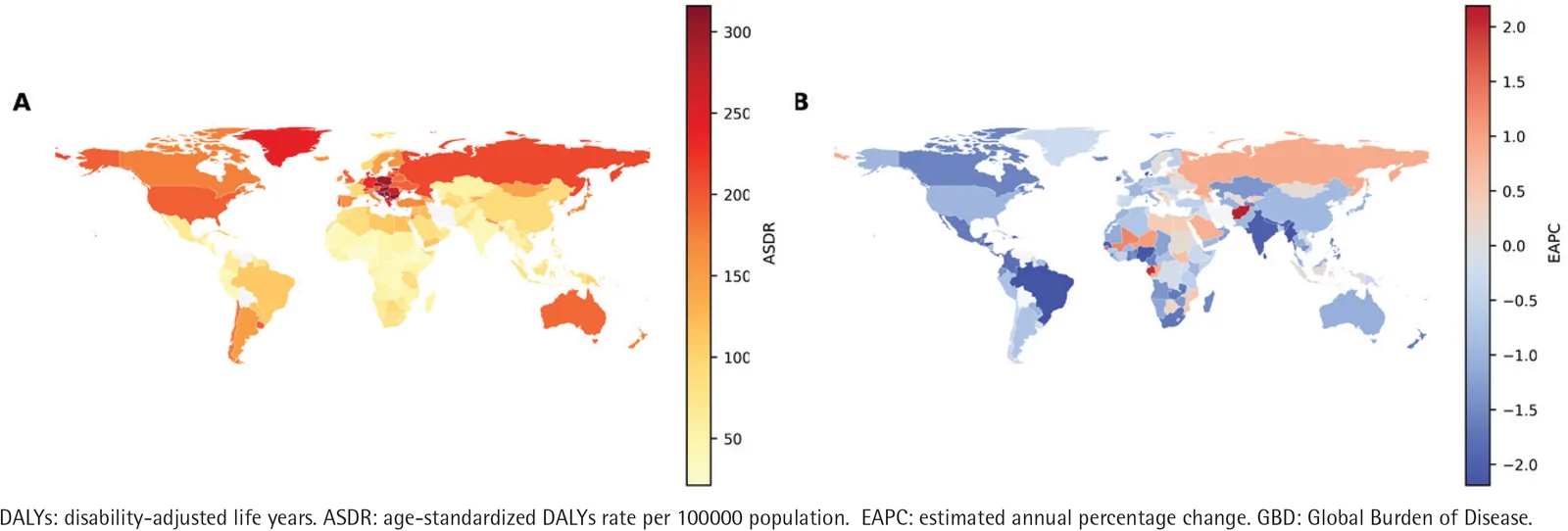
Geographical distribution of ASDR and temporal change for smoking-attributable musculoskeletal disorders across 204 countries and territories in a secondary dataset analysis of GBD 2021 estimates: A) 2021 ASDR; B) EAPC in ASDR from 1990 to 2021

In 2021, the highest national ASDRs were observed in Montenegro (368.35), Bulgaria (353.08) Serbia (349.78), whereas the lowest ASDRs were observed in Nigeria (21.23), Ghana (24.98), and Ethiopia (27.15) (Figure 2; and Supplementary file Table S4). Country-level EAPC values ranged from -3.15% in Panama to 2.52% in Afghanistan, showing substantial heterogeneity in temporal change in ASDR (Figure 2; and Supplementary file Figure S1).

### Age-specific distribution

**Figure 3:**
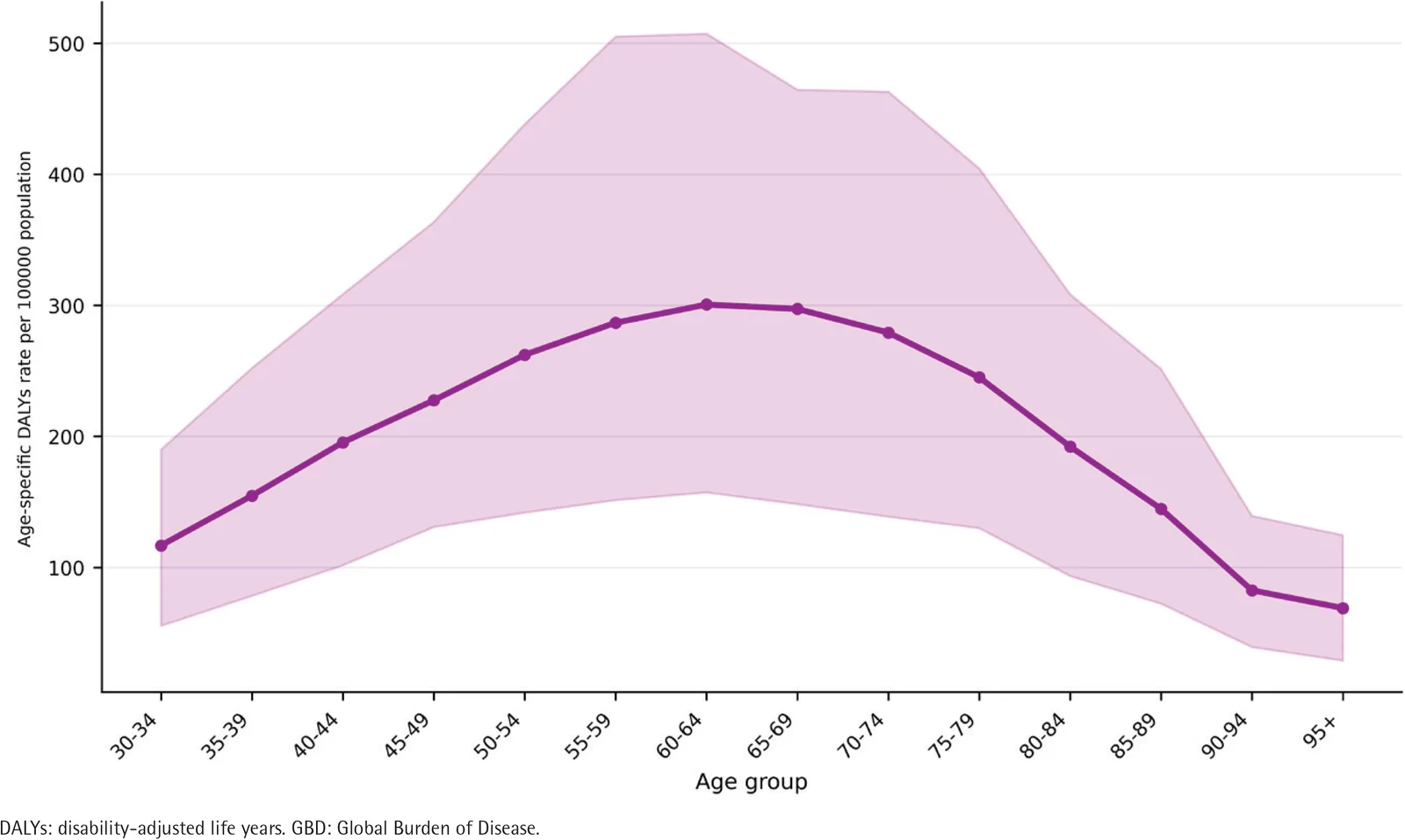
Global age-specific DALYs rate per 1000000 population with 95% uncertainty intervals, for both sexes combined, for smoking-attributable musculoskeletal disorders in a secondary dataset analysis of GBD 2021 estimates, 2021

The global age-specific DALYS rate increased from early adulthood and reached its maximum at 60–64 years, with a rate of 300.61 (95% UI: 157.31–507.38) in 2021 (Figure 3). Full country-by-age heat maps showed similar adult-age concentration patterns but substantial cross-national heterogeneity in the magnitude of rates (Supplementary file Figure S2).

### Joinpoint trend analysis

**Figure 4:**
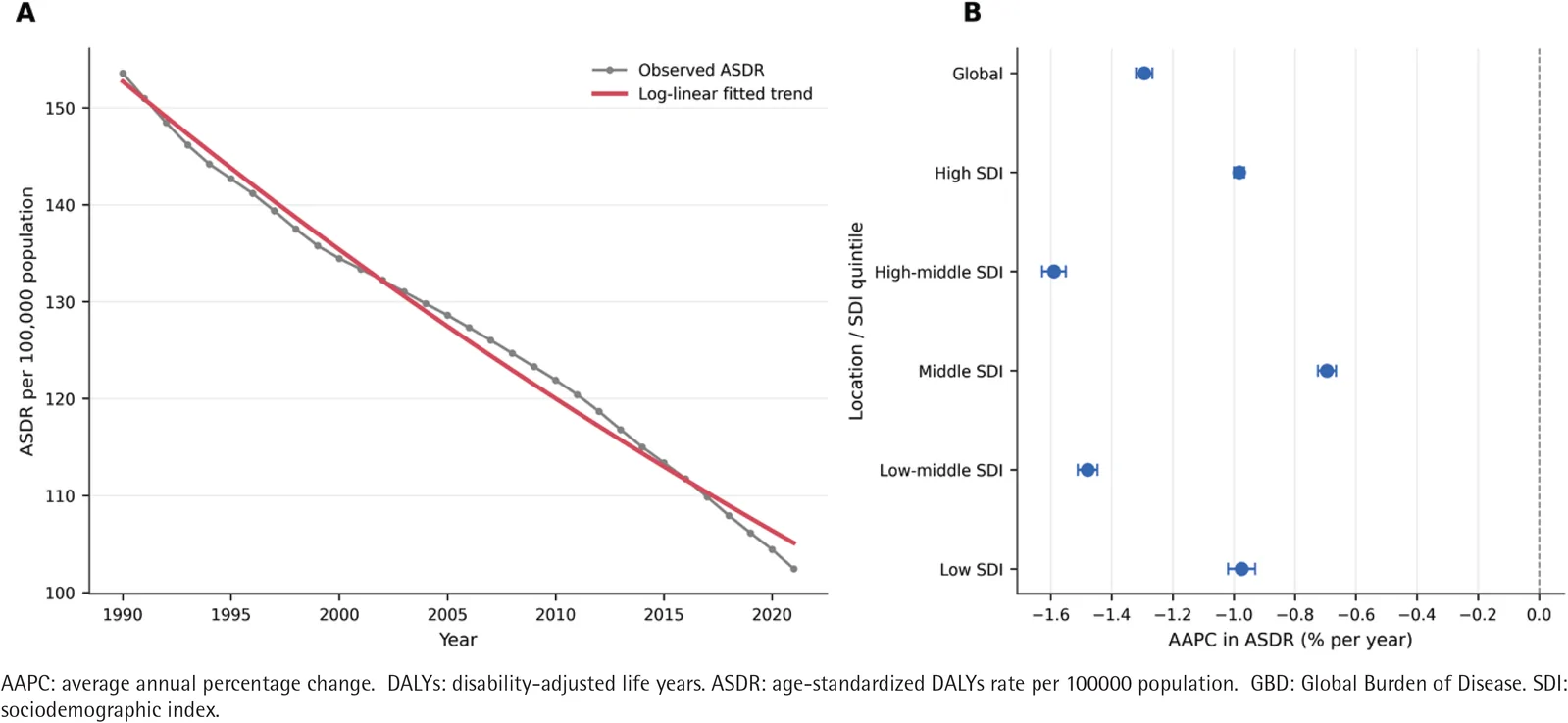
Joinpoint analysis of global and SDI-stratified ASDR trends for smoking-attributable musculoskeletal disorders in a secondary dataset analysis of GBD 2021 estimates, 1990–2021: A) observed and fitted global ASDR; B) AAPC estimates with 95% confidence intervals for the global and SDI-specific series

Joinpoint regression showed a negative AAPC in the global series and in all SDI quintiles over 1990–2021 (Table 2 and Figure 4). The steepest overall ASDR decline was observed in High-middle SDI regions (AAPC= -1.59%; 95% CI: -1.63 – -1.55), whereas the smallest decline was observed in Middle SDI regions (AAPC= -0.69%; 95% CI: -0.72 – -0.67) (Table 2). Segment-specific APCs for the global and SDI-specific series are provided in Supplementary file Table S2 and Table S2 and Supplementary file Figure S3.

**Table 2:** Joinpoint-based average annual percentage change in ASDR, for both sexes combined, for smokingattributable musculoskeletal disorders in a secondary dataset analysis of GBD 2021 estimates, 1990–2021

*Location*	*AAPC*	*95% LCI*	*95% UCI*	*p*
**Global SDI region**	-1.2934	-1.3198	-1.2669	<0.001
**SDI region**				
High	-0.9829	-1.0003	-0.9655	<0.001
High-middle	-1.5891	-1.6278	-1.5503	<0.001
Middle	-0.6950	-0.7247	-0.6652	<0.001
Low-middle	-1.4786	-1.5110	-1.4462	<0.001
Low	-0.9745	-1.0191	-0.9298	<0.001

DALYs: disability-adjusted life years; ASDR: age-standardized DALYS rate per 100000 population. SDI: sociodemographic index. GBD: Global Burden of Disease. AAPC: average annual percentage change. LCI: lower confidence interval. UCI: upper confidence interval.

### SDI-stratified and regional burden patterns

In 2021, High-SDI regions had the highest ASDR at 154.13 (95% UI: 81.19–231.32), followed by Middle-SDI regions at 84.43 (95% UI: 46.03–128.32) and High-middle-SDI regions at 70.86 (95% UI: 39.25–108.41) (Table 1; and Supplementary file Figure S4). Absolute DALYs increased in all SDI quintiles between 1990 and 2021, with the largest relative increases in Middle-SDI regions (89.15%) and Low-SDI regions (71.71%) (Table 1; and Supplementary file Figure S4).

Across GBD regions in 2021, Central Europe had an ASDR of 303.04 (95% UI: 161.72–455.38), Eastern Europe 206.15 (95% UI: 109.59–308.17), and Western Europe 195.09 (95% UI: 102.22–293.24) (Supplementary file Table S1). Annual SDI-stratified trend curves are shown in Supplementary file Figure S5.

### Frontier and socioeconomic inequality analyses

In the 2021 frontier analysis, several countries had an observed ASDR well above the estimated frontier at comparable SDI levels, indicating a visible distance between observed burden and the frontier estimate (Supplementary file Figure S6).

Regression-based inequality analysis showed a positive SDI-related slope in both 1990 and 2021, and concentration curves indicated that DALYs remained disproportionately concentrated among populations with higher SDI ranks (Supplementary file Figure S7).

### Exploratory projections to 2050

ARIMA projections suggested that global DALYs may decrease from 8.84 million (95% UI: 8.82–8.86) in 2022 to 7.53 million (95% UI: 4.12–10.93) in 2050, corresponding to a relative change of -14.8% (Table 3; and Supplementary file Figure S9). Global ASDR was projected to decrease from 100.23 (95% UI: 99.96–100.51) in 2022 to 45.40 (95% UI: 29.06–61.74) in 2050, corresponding to a relative change of -54.7% (Table 3; and Supplementary file Figure S8). The BAPC sensitivity analysis for global DALYs yielded the same direction of change, with a projected decrease from 8.83 million (95% UI: 8.63–9.04) in 2022 to 7.22 million (95% UI: 1.98–12.47) in 2050 (Table 3).

**Table 3:** Exploratory global projections of smoking-attributable musculoskeletal disorder burden in a secondary dataset analysis of GBD 2021 estimates, 2022–2050

*Series*	*2022*	*2050*	*Percent change*	*Interpretation*
**ARIMA** Global DALYs (million) (95% UI)	8.84 (8.82–8.86)	7.53 (4.12–10.93)	-14.8	Primary exploratory number forecast
**BAPC** Global DALYs (million) (95% UI)	8.83 (8.63–9.04)	7.22 (1.98–12.47)	-18.2	Sensitivity analysis; direction consistent with ARIMA
**ARIMA** Global ASDR	100.23 (99.96–100.51)	45.40 (29.06–61.74)	-54.7	Exploratory ASDR projection

DALYs: disability-adjusted life years; ASDR: age-standardized DALYS rate per 100000 population. GBD: Global Burden of Disease. ARIMA: autoregressive integrated moving average. BAPC: Bayesian age-period-cohort.

## DISCUSSION

This secondary dataset analysis of GBD 2021 estimates showed that smoking-attributable MSDs remain a substantial component of global disability burden. The principal pattern was a divergence between declining ASDR and persistent absolute DALYs: global ASDR decreased from 1990 to 2021, but all-age DALYs increased. This divergence is compatible with evidence linking cigarette exposure to impaired bone health^21^. It is also consistent with musculoskeletal burden projections showing that absolute needs may persist even when age-standardized rates decline^22^.

Several findings extend the public health interpretation of smoking-attributable burden of MSDs. High-SDI regions continued to carry the highest age-standardized burden in 2021, while relative growth in DALYs was most pronounced in Middle-SDI and Low-SDI regions. From a healthy-ageing perspective, this pattern is relevant because functional limitation, pain, and rehabilitation needs accumulate across the life course^23^. Equity concerns are also important because demographic transition may shift absolute disability needs toward settings with fewer health-system resources^24^. These concerns are reinforced by evidence on tobacco industry conduct in low-income and middle-income countries^25^. At the same time, the decline in ASDR is consistent with the potential population benefit of strengthened tobacco-control policies^26^.

The frontier and inequality analyses also require cautious interpretation. Effective differences from the frontier indicate the distance between observed ASDR and the estimated lowest attainable ASDR at a comparable level of SDI, but they do not identify the causes of that distance. Similarly, the concentration-curve and slope-based analyses summarize socioeconomic distribution of burden across countries, not within-country inequalities. These results may help identify where future descriptive and policyfocused studies could be prioritized, but they should not be interpreted as direct evidence that specific health-system or tobacco-control interventions caused the observed patterns.

The exploratory projections to 2050 suggest continued declines in global ASDR and a slower reduction in DALYs. These projections should be interpreted as trend-continuation estimates rather than forecasts that incorporate future changes in smoking behavior, tobacco-control policies, healthcare access, or emerging nicotine products. Comparable smoking-attributable forecasting work has been reported for lower respiratory infections^27^. Related trend-and-forecast approaches have also been applied to knee osteoarthritis attributable to high body-mass index^28^. Other recent burden studies have used long-horizon forecasting for air-pollution-related neoplasms^29^. Similar projection frameworks have been reported for abdominal wall hernias^30^. Long-horizon projections have also been applied to cancer burden analyses^31^.

### Strengths and limitations

This study has several strengths, including the use of standardized GBD 2021 estimates, multi-level reporting across global, regional, national, and SDI strata, and combined assessment of trends, inequality, frontier performance, and projections. Several limitations should also be acknowledged. First, all estimates derive from modeled GBD outputs and therefore inherit the assumptions, input-data quality, and uncertainty of the underlying framework, particularly in data-sparse settings. Second, the ecological design precludes causal inference at the individual level. Third, the available data package did not support disease-specific smoking-attributable subtyping within the broader category of MSDs. Fourth, long-term projections become increasingly uncertain over time and should be interpreted as scenario-like extrapolations rather than precise future observations. Clinical interpretation also requires caution because smoking has been linked to lower bone-mineral-density^32^ and pain-related mechanisms^33^. Rehabilitation and physical activity remain important considerations for people already living with chronic musculoskeletal symptoms^34^. Finally, the emergence of alternative nicotine-delivery products underscores the need for additional studies on musculoskeletal outcomes rather than direct extrapolation from combustible cigarette evidence^35^.

## CONCLUSIONS

Smoking-attributable MSDs remain a measurable source of global disability. From 1990 to 2021, global ASDR declined while the absolute number of DALYs increased, and high age-standardized burden remained concentrated in several higher SDI and European settings. These findings may warrant greater consideration of musculoskeletal outcomes in future tobacco-related burden assessment and rehabilitation planning. Additional disease-specific and policy-oriented studies are needed before stronger causal or health-system recommendations can be made.

## Supplementary Material



## Data Availability

All data used in this study are publicly available from the Global Health Data Exchange and the GBD Results Tool. Processed data and analysis outputs can be made available from the corresponding author upon reasonable request.
