# Supplementary material for: Smoking-attributable burden of musculoskeletal disorders, 1990–2021, with exploratory projections to 2050: A secondary dataset analysis of the Global Burden of Disease Study 2021
Source: Tob Induc Dis. 2026 Aug 25;24:10.18332/tid/226288. doi: 10.18332/tid/226288 (PMC13520598; doi:10.18332/tid/226288)
Supplement: Supplementary file 1 [file TID-24-148-s1.pdf]

Tobacco Induced Diseases

Supplementary file

© 2026 Fu Z. et al.

DOI:
10.18332/tpc/226288

The content has been provided by the author(s) and has not been reviewed, verified, or endorsed by European Publishing. It may not have undergone peer review. The views, opinions, and recommendations expressed are solely those of the author(s) and do not necessarily reflect the position of European Publishing. European Publishing accepts no responsibility or liability for any consequences arising from the use of, or reliance on, this content.

Supplementary Tables

Smoking-attributable musculoskeletal disorders

Supplementary Table S1

Supplementary Table S1. Detailed global, SDI, and regional burden of smoking-attributable musculoskeletal disorders in a secondary dataset analysis of GBD 2021 estimates, 1990 and 2021.

Location	DALYs 1990 (95% UI)	DALYs 2021 (95% UI)	Change (%)	ASDR 1990 (95% UI)	ASDR 2021 (95% UI)	EAPC (95% CI)
Global	6817893.48(3846047.12-10121030.78)	8885766.69(4720131.54-13678166.55)	30.33%	153.57(85.94-228.28)	102.44(54.6-157.3)	-1.2 (-1.24 - -1.16)
SDI quintiles						
Low SDI	390610.52(206987.1-606886.9)	670714.5(368154.6-1070987.32)	71.71%	85.32(44.87-131.92)	63.16(34.35-101.01)	-0.94 (-1.03 - -0.85)
Low-middle SDI	419767.85(218888.52-648822.1)	560440.55(296842.02-920138.75)	33.51%	88.11(46.03-136.61)	55.58(29.64-91.59)	-1.37 (-1.48 - -1.25)
Middle SDI	427147.97(243367.29-639866.16)	807931.88(444894.05-1223177.64)	89.15%	104.78(59.1-157.65)	84.43(46.03-128.32)	-0.64 (-0.7 - -0.57)
High-middle SDI	1008721.69(583491.96-1522282.89)	1386579.57(766273.85-2121313.93)	37.46%	116.65(67.25-178.02)	70.86(39.25-108.41)	-1.49 (-1.52 - -1.47)
High SDI	4560698.14(2602809.47-6725599.17)	5447974.89(2822817.57-8310741.63)	19.45%	209.92(120.23-309.62)	154.13(81.19-231.32)	-0.88 (-0.91 - -0.85)
GBD regions						
Andean Latin America	13436.23(7039.55-21766.96)	27571.88(14217.7-46071.77)	105.21%	54.08(28.21-86.81)	42.28(21.79-71.12)	-0.79 (-0.81 - -0.76)
Australasia	61145.95(32917.47-91971.21)	75905.27(37441.03-116703.06)	24.14%	271.26(146.46-405.39)	187.33(91.78-289.52)	-1.16 (-1.22 - -1.1)
Caribbean	28215.67(15090.06-44846.02)	39848.37(21270.52-66418)	41.23%	99.99(53.55-156.58)	74.9(39.98-125.2)	-1.1 (-1.23 - -0.98)
Central Asia	63604.86(35203.1-97749.93)	109446.26(61501.54-172567.34)	72.07%	119.75(65.55-180.48)	111.12(62.08-174.51)	-0.09 (-0.24 - 0.06)
Central Europe	505346.27(293557.8-	509841.03(266324.18-	0.89%	346.73(201.93-	303.04(161.72-	-0.42 (-0.45 - -

	771863.94)	778526.63)		526.57)	455.38)	0.39)
Central Latin America	111711.36(62306.31-175973.02)	168346.74(89989.26-275912.17)	50.70%	104.56(58.16-165.07)	62.54(33.34-102.82)	-1.72 (-1.8 - -1.65)
Central Sub-Saharan Africa	15158.43(7664.54-24919.97)	34812.75(18141.74-58755.39)	129.66%	49.87(25.27-82.49)	42.29(22.15-71.64)	-0.37 (-0.48 - -0.27)
East Asia	1340230.97(797520.65-2028748.53)	1937243.92(1103808.22-2919399.3)	44.55%	130.18(76.72-197.22)	91.2(51.58-135.67)	-0.9 (-0.98 - -0.82)
Eastern Europe	490796.26(284265-741183.77)	627776.8(326424.86-951781.47)	27.91%	183.84(106-273.98)	206.15(109.59-308.17)	0.62 (0.46 - 0.77)
Eastern Sub-Saharan Africa	57212.09(29802.61-89834.77)	123290(66454.3-199789.58)	115.50%	59.46(30.82-92.43)	52.01(28.07-84.28)	-0.45 (-0.46 - -0.43)
High-income Asia Pacific	461087.1(259460.85-684199.74)	442026.5(236349.23-708673.06)	-4.13%	226.57(127.43-334.92)	160.75(85.54-257.33)	-1.08 (-1.15 - -1.02)
High-income North America	886040.31(474994.59-1289636.4)	949035.49(467546.01-1444485.56)	7.11%	279.31(150.5-403.34)	193.91(94.96-290.4)	-1.03 (-1.08 - -0.98)
North Africa and Middle East	269310.27(147354.64-415967.73)	659320.93(374522.93-1036578.57)	144.82%	123.55(66.76-188.66)	111.1(63.34-174.11)	-0.32 (-0.36 - -0.28)
Oceania	4624.2(2431.21-7149.34)	10870.75(5755.21-17178.38)	135.08%	111.26(58.78-171.19)	99.83(52.74-157.66)	-0.44 (-0.5 - -0.38)
South Asia	640290.58(339894.81-1003524.76)	841662.11(443853.96-1354043.93)	31.45%	90.36(48.19-140.8)	51.66(27.62-84.1)	-1.68 (-1.77 - -1.58)
Southeast Asia	283215.05(162334.07-426342.14)	539799.68(290587.81-806241.84)	90.60%	89.31(50.28-134.75)	71.89(38.81-107.34)	-0.7 (-0.75 - -0.65)
Southern Latin America	93006.42(51398.25-145020.83)	128810.94(65277.93-207063.48)	38.50%	197.11(109.01-306.35)	160.93(81.29-256.59)	-0.61 (-0.72 - -0.5)
Southern Sub-Saharan Africa	39691.79(21745.29-61095.43)	57518.06(31545.93-89658.05)	44.91%	121.18(64.77-188.9)	76(41.95-119.02)	-1.57 (-1.78 - -1.37)
Tropical Latin America	229828(132363.52-348176.12)	289630.51(145574.49-455034.77)	26.02%	199.51(113.12-306.31)	111.01(55.74-174.09)	-2.17 (-2.28 - -2.05)
Western Europe	1173170.33(644167.87-1734488.07)	1227010.32(636060.67-1884230.44)	4.59%	247.94(137.84-365.28)	195.09(102.22-293.24)	-0.68 (-0.7 - -0.65)
Western Sub-Saharan Africa	50771.36(25022.35-85124.63)	85998.39(42946.79-149634.95)	69.38%	46.16(22.73-76.28)	30.61(15.19-53.5)	-1.4 (-1.53 - -1.27)
African Region	177547.07(93346.67-276554.69)	336092.64(179365.86-553162.69)	89.30%	63.56(33.08-98.5)	47.73(25.61-78.26)	-0.96 (-0.97 - -0.94)
Central Africa	18269.09(9293.21-29401.09)	38943.77(20118.53-66435.19)	113.17%	49.49(25.07-79.79)	40.06(20.85-68.51)	-0.57 (-0.68 - -0.45)
Central Europe, Eastern Europe, and	1059747.39(617232.67-1611444.01)	1247064.09(654650.61-1906756.23)	17.68%	227.45(132.76-343.95)	216.9(116.01-330.07)	-0.01 (-0.11 - 0.09)

Central Asia						
Commonwealth High Income	345628.77(185355.84-507382.55)	364846.29(184288.5-556495.59)	5.56%	263.23(142.58-383.79)	179.37(93.39-268.6)	-1.22 (-1.26 - -1.18)
Commonwealth Low Income	18304.22(9338.14-28809.93)	37534.54(20288.8-60849.05)	105.06%	63.74(32.49-100.67)	58.16(31.52-94.6)	-0.28 (-0.47 - -0.09)
Commonwealth Middle Income	720370.85(378148.58-1130201.11)	988454.35(524149.47-1609546.49)	37.21%	84.52(44.54-132.25)	49.77(26.72-81.54)	-1.6 (-1.72 - -1.49)
East Asia & Pacific - WB	2141037.03(1265889.61-3217886.2)	2998077.85(1663034.66-4479873.04)	40.03%	135.83(80.19-204.9)	93.8(52.47-141.1)	-1.02 (-1.07 - -0.98)
Eastern Africa	52744.42(27490.36-82475.13)	117728.36(63366.78-192197.69)	123.21%	58.56(30.4-91.17)	51.96(28.09-85.01)	-0.41 (-0.43 - -0.39)
Eastern Mediterranean Region	231083.44(124109.94-358318.98)	570697.36(309142.74-918003.47)	146.97%	103.3(55.15-158.61)	93.81(51.13-150.14)	-0.27 (-0.33 - -0.2)
Europe & Central Asia - WB	2297793.3(1311264.63-3417839.55)	2608311.41(1355027.52-3997157.05)	13.51%	236.23(136.17-354.68)	203.68(108.39-307.33)	-0.36 (-0.42 - -0.3)
European Region	2309132.03(1318139.98-3435709.48)	2626026.49(1364115.46-4028724.28)	13.72%	236.18(136.18-354.67)	203.34(108.19-307.15)	-0.37 (-0.42 - -0.31)
High-income	2674450.11(1465122.6-3972918.01)	2822788.52(1440372.2-4302089.37)	5.55%	251.74(139.03-369.31)	186.58(94.79-278.97)	-0.87 (-0.9 - -0.85)
Latin America & Caribbean - WB	475177.8(259404.94-733603.81)	652784.33(330309.09-1054812.03)	37.38%	147.17(79.54-227.14)	89.86(45.51-145.28)	-1.74 (-1.82 - -1.66)
Latin America and Caribbean	383191.26(213252.52-587897.59)	525397.5(269250.32-848973.78)	37.11%	139.15(75.96-216.26)	81.26(41.62-131.48)	-1.92 (-2.01 - -1.84)
Middle East & North Africa - WB	185352.91(99762.38-288511.67)	485745.44(268807.56-762184.84)	162.07%	116.03(61.95-178.06)	106.3(59.2-168.45)	-0.24 (-0.3 - -0.18)
North America	885947.89(474943.41-1289520.28)	948924.4(467473.81-1444333.18)	7.11%	279.27(150.48-403.29)	193.88(94.95-290.36)	-1.03 (-1.08 - -0.98)
Northern Africa	83768.24(45970.98-128391.24)	196263.42(112090.82-300430.33)	134.29%	106.72(57.99-162.24)	100.84(57.71-154.29)	-0.15 (-0.22 - -0.08)
Region of the Americas	1358082.7(743747.49-2019149.45)	1597760.58(797901.35-2452534.48)	17.65%	212.19(115.86-315.79)	130.61(64.96-199.47)	-1.55 (-1.57 - -1.52)
South Asia - WB	651972.88(345734.2-1022222.58)	862344.82(455199.08-1390323.97)	32.27%	89.36(47.6-139.32)	51.5(27.55-83.89)	-1.65 (-1.79 - -1.52)
South-East Asia Region	668496.88(358574.46-1034529.44)	853756.21(446344.91-1333178.79)	27.71%	88.8(47.52-137.16)	50.09(26.5-78.6)	-1.74 (-1.86 - -1.61)
Southeast Asia, East Asia, and Oceania	1628070.22(963504.6-2458167.49)	2487914.35(1395386.38-3701914.11)	52.81%	120.64(71.04-181.68)	86.35(48.69-127.76)	-0.87 (-0.93 - -0.81)
Southern Africa	53867.85(29043.04-83594.8)	86435.57(47659.61-133492.44)	60.46%	99.3(52.96-155.95)	66.93(36.69-103.68)	-1.32 (-1.41 - -1.23)

Sub-Saharan Africa	162833.66(85176.87-254525.98)	301619.2(160356.25-500254.49)	85.23%	60.76(31.48-94.46)	44.97(24.07-74.04)	-1.01 (-1.03 - -0.99)
Sub-Saharan Africa - WB	169640.78(88613.71-265403.58)	317427.33(168457.31-527714.53)	87.12%	60.49(31.31-94.02)	45.3(24.2-74.8)	-0.97 (-0.99 - -0.95)
Western Africa	44271.54(21696.65-75027.85)	74100.5(36679.4-129016.36)	67.38%	44.5(21.76-74.58)	29.47(14.54-51.64)	-1.41 (-1.53 - -1.29)
Western Pacific Region	2034862.45(1206160.97-3066159.99)	2838795.41(1572962.49-4235526.95)	39.51%	138.86(82.22-210.02)	95.53(53.39-143.67)	-1.02 (-1.07 - -0.98)
World Bank High Income	3418306.89(1906968.01-5052547.72)	3744559.91(1913901.83-5713708.19)	9.54%	246.36(138.5-363.6)	191.73(99-288.37)	-0.71 (-0.75 - -0.68)
World Bank Low Income	102588.53(53786.42-159361.81)	217476.99(114556.94-357272.66)	111.99%	60.71(31.65-93.6)	54.57(29.04-89.68)	-0.32 (-0.41 - -0.24)
World Bank Lower Middle Income	951108.27(502112.66-1477135.86)	1450132.6(777052.34-2345266.24)	52.47%	88.96(46.76-137.88)	57.33(31.04-92.83)	-1.36 (-1.46 - -1.25)
World Bank Upper Middle Income	2334918.9(1362058.99-3538926.88)	3461446.08(1915675.19-5212713)	48.25%	134.51(78.29-205.32)	95.61(53.19-145.17)	-0.96 (-1 - -0.93)

Note: DALY = disability-adjusted life year; ASDR = age-standardized DALY rate per 100,000 population; SDI = Socio-demographic Index; GBD = Global Burden of Disease; UI = uncertainty interval; CI = confidence interval; EAPC = estimated annual percentage change. Values are for both sexes combined.

Supplementary Table S2

Supplementary Table S2. Segment-specific annual percentage changes in age-standardized DALY rates for smoking-attributable musculoskeletal disorders from joinpoint regression in a secondary dataset analysis of GBD 2021 estimates, 1990-2021.

Location	Range	APC	95%LCI	95%UCI	p
Global	1990-1993	-1.662	-1.7472	-1.5768	<0.001
	1993-1996	-1.1336	-1.3076	-0.9593	<0.001
	1996-2006	-1.2769	-1.4487	-1.1048	<0.001
	2006-2011	-0.8979	-0.9266	-0.8692	<0.001
	2011-2016	-1.1222	-1.1755	-1.0689	<0.001
	2016-2021	-1.4995	-1.554	-1.445	<0.001
High SDI	1990-1994	-1.5198	-1.573	-1.4665	<0.001
	1994-1999	-0.8648	-0.9196	-0.81	<0.001
	1999-2006	-0.6996	-0.7284	-0.6708	<0.001
	2006-2011	-0.7916	-0.8448	-0.7384	<0.001
	2011-2016	-0.9892	-1.0432	-0.9351	<0.001
	2016-2021	-1.2505	-1.2901	-1.2109	<0.001
High-middle SDI	1990-1993	-2.2514	-2.3785	-2.1242	<0.001
	1993-1996	-1.4644	-1.722	-1.2061	<0.001
	1996-1999	-1.8859	-2.1377	-1.6335	<0.001
	1999-2009	-1.3191	-1.3417	-1.2965	<0.001
	2009-2019	-1.5056	-1.529	-1.4822	<0.001
	2019-2021	-2.097	-2.3594	-1.834	<0.001
Middle SDI	1990-1993	-0.7741	-0.8636	-0.6845	<0.001
	1993-1999	-0.4125	-0.453	-0.3719	<0.001
	1999-2005	-0.237	-0.2756	-0.1983	<0.001
	2005-2008	-0.4892	-0.6651	-0.3129	<0.001
	2008-2011	-0.7738	-0.9481	-0.5992	<0.001
	2011-2014	-1.4326	-1.6095	-1.2554	<0.001
	2014-2021	-1.031	-1.0554	-1.0065	<0.001
Low-middle SDI	1990-1996	-0.9782	-1.0115	-0.9449	<0.001
	1996-1999	-2.4522	-2.6475	-2.2566	<0.001

	1999-2007	-0.494	-0.5199	-0.4681	<0.001
	2007-2011	-1.109	-1.207	-1.0108	<0.001
	2011-2016	-2.4581	-2.5203	-2.3959	<0.001
	2016-2019	-2.7155	-2.9195	-2.511	<0.001
	2019-2021	-1.8367	-2.0462	-1.6267	<0.001
Low SDI	1990-1996	-0.3178	-0.3657	-0.2698	<0.001
	1996-1999	-1.4596	-1.7394	-1.179	<0.001
	1999-2006	-0.3131	-0.3603	-0.2658	<0.001
	2006-2011	-0.9747	-1.063	-0.8862	<0.001
	2011-2016	-1.6438	-1.7319	-1.5556	<0.001
	2016-2019	-2.0377	-2.3192	-1.7553	<0.001
	2019-2021	-1.2328	-1.5239	-0.9408	<0.001

Note: APC = annual percentage change; ASDR = age-standardized DALY rate per 100,000 population; DALY = disability-adjusted life year; SDI = Socio-demographic Index; GBD = Global Burden of Disease; LCI = lower confidence interval; UCI = upper confidence interval.

Supplementary Table S3

Supplementary Table S3. ARIMA model parameters and residual diagnostics for smoking-attributable musculoskeletal disorder burden estimates in a secondary dataset analysis of GBD 2021 estimates, 1990-2021.

Measure	Location	ARIMA model	χ2	AIC	Ljung-Box Q	RMSE	MAPE
ASDR	Global	1,1,1	0.04	-27.13	0.85	0.13	8.04%
	High SDI	0,2,1	1.26	-17.66	0.26	0.16	16.58%
	High-middle SDI	0,1,4	0.07	-19.5	0.79	0.13	13.22%
	Middle SDI	0,2,1	0.94	-52.02	0.33	0.09	7.82%
	Low-middle SDI	0,2,1	0.1	-1.39	0.75	0.21	15.35%
	Low SDI	4,1,1	0.01	-20.12	0.92	0.13	5.69%
Number of DALYs cases	Global	0,2,1	0.51	644.13	0.48	10026.52	9.77%
	High SDI	0,2,1	0.43	612.58	0.51	5952.60	8.54%
	High-middle SDI	3,1,0	0.35	608	0.55	3570.05	15.11%
	Middle SDI	1,1,1	0.13	515.38	0.72	818.55	10.86%
	Low-middle SDI	0,1,4	0.2	532.39	0.65	953.61	14.25%
	Low SDI	2,1,0	0.95	532.59	0.33	1083.30	16.14%

Note: ARIMA = autoregressive integrated moving average; AIC = Akaike information criterion; RMSE = root mean square error; MAPE = mean absolute percentage error; ASDR = age-standardized DALY rate per 100,000 population; DALY = disability-adjusted life year; GBD = Global Burden of Disease. Diagnostics were based on 1990-2021 observed series.

Supplementary Table S4

Supplementary Table S4. Country-level DALY burden and age-standardized DALY rates of smoking-attributable musculoskeletal disorders in a secondary dataset analysis of GBD 2021 estimates, 1990 and 2021.

Countries	DALY number (95% UIs)	DALY number (95% UIs)	Percentage change(100%)	DALY number (95% UIs)	DALY number (95% UIs)	EAPC (95% CIs)
Countries	1990	2021	Percentage change(100%)	1990	2021	EAPC (95% CIs)
Afghanistan	2113.47(986.6-3740.33)	10636.58(5262.39-18062.15)	403.28%	29.42(13.74-52.24)	55.22(27.29-94.35)	2.52 (2.35 - 2.68)
Albania	6237.51(3382.7-9450.34)	9811.12(5351.25-15500.2)	57.29%	242.52(128.08-362.9)	265.69(149.57-422.87)	0.45 (0.36 - 0.55)
Algeria	16319.63(8652.68-25125.75)	39208.25(21277.39-61359.08)	140.25%	110.16(57.98-166.66)	89.51(48.23-140.54)	-0.71 (-0.76 - -0.66)
American Samoa	37.06(19.5-59.09)	48.83(24.52-79.05)	31.76%	113.31(59.27-180.22)	91.97(46.19-149.3)	-0.82 (-0.95 - -0.69)
Andorra	151.26(81.65-226.27)	277.87(153.31-439.62)	83.70%	238.37(129.73-358.43)	215.39(119-339.25)	-0.32 (-0.33 - -0.3)
Angola	4618.44(2353.73-7647.09)	10157.17(5258.2-17316.44)	119.93%	83.27(42.74-137.36)	53.72(27.72-92.4)	-1.38 (-1.46 - -1.29)
Antigua and Barbuda	30.61(14.04-57.81)	74.65(35.18-135.42)	143.87%	60.65(28.1-114.36)	65.82(30.96-119.7)	0.2 (0.18 - 0.23)
Argentina	62323.5(33807.72-98171.38)	80003.62(42019.28-131907.72)	28.37%	193.9(105.43-304.8)	151.59(80.03-248.65)	-0.74 (-0.86 - -0.62)
Armenia	5201.49(3010.72-7783.83)	6555.48(3639.05-9896.8)	26.03%	166.74(96.3-248.55)	158.1(89.26-238.71)	-0.07 (-0.11 - -0.02)
Australia	50709.37(27444.35-76261.64)	64595.16(31640.23-100241.53)	27.38%	269.23(146.15-402.41)	190.2(92.49-296.28)	-1.08 (-1.15 - -1.01)
Austria	18915.76(10320.56-29687.82)	25108.52(13777.68-38134.83)	32.74%	201.71(111.57-308.22)	193.6(105.23-293.45)	0.05 (-0.04 - 0.14)
Azerbaijan	6654.36(3566.11-9697.23)	15711.29(9121.75-24275.35)	136.11%	114.82(61.94-167.5)	129.51(75.52-200.52)	0.54 (0.38 - 0.7)
Bahamas	86.09(41.09-148.79)	184.18(88.13-348)	113.94%	43.39(20.8-74.49)	38.98(18.45-73.87)	-0.38 (-0.45 - -0.3)
Bahrain	427.07(213.98-680.25)	1826.51(985.39-2978.55)	327.68%	116.57(58.43-176.85)	104.57(55.86-168.95)	-0.25 (-0.4 - -0.11)
Bangladesh	81213.52(45080.81-121127.27)	160140.2(89008.41-246341.05)	97.18%	141.52(78.22-212.18)	112.54(62.57-171.41)	-0.66 (-0.75 - -0.57)

Barbados	131.61(64.55-229.16)	174.56(83.02-336.08)	32.63%	52.31(26.02-91.69)	42.92(20.03-83.39)	-0.97 (-1.13 - -0.81)
Belarus	22808.49(12969.23-34979.17)	25476.15(14103.43-40084.12)	11.70%	188.5(106.66-284.31)	185.33(100.82-289.68)	0.08 (-0.04 - 0.2)
Belgium	32405.81(18106.98-48995.22)	30856.46(14602.94-47632.81)	-4.78%	256.44(145.39-385.93)	193.2(90.97-296.96)	-0.92 (-0.97 - -0.86)
Belize	72.14(36.25-120.18)	219.31(108.39-379.86)	204.01%	69.19(35.11-114.79)	62.16(30.7-106.48)	-0.12 (-0.24 - -0.01)
Benin	1158.6(551.86-1889.21)	2215.16(1071.14-3751.06)	91.19%	47.69(23.44-77.61)	31.02(15.01-52.76)	-1.69 (-1.96 - -1.42)
Bermuda	64.71(32.81-103.73)	74.16(38.26-134.07)	14.60%	94.12(48.53-150.9)	73.48(37.65-133.17)	-1.03 (-1.15 - -0.91)
Bhutan	250.07(116.58-429.82)	437.78(214.25-803.2)	75.06%	75.33(35.24-127.21)	60.82(29.74-110.89)	-0.75 (-0.79 - -0.72)
Bolivia (Plurinational State of)	1794.76(826.02-2964.34)	4899.5(2564.01-8195.26)	172.99%	45.18(21.22-73.88)	45.16(23.7-76.33)	0.52 (0.25 - 0.79)
Bosnia and Herzegovina	12800.14(7199.3-19699.91)	15180.28(8057.17-23380.01)	18.59%	269.46(150.34-414.81)	317.65(168.95-475.13)	0.78 (0.57 - 0.98)
Botswana	647.68(341.63-1003.12)	1879.74(1001.77-3036)	190.23%	90.46(48.43-141.36)	95.58(51.51-153.54)	0.25 (0.22 - 0.27)
Brazil	225972.59(130276.92-342856.38)	282866.62(142253.07-444493.69)	25.18%	200.74(114.07-308.74)	110.95(55.73-174.29)	-2.19 (-2.31 - -2.07)
Brunei Darussalam	308.22(165.46-488.09)	586.96(298.57-975.67)	90.44%	170.87(92.17-261.38)	114.23(58.2-190.17)	-1.34 (-1.47 - -1.21)
Bulgaria	37392.99(20692.99-57606.06)	36752.45(19843.71-54869.3)	-1.71%	342.39(191.36-520.21)	353.08(193.93-519.01)	0.01 (-0.05 - 0.07)
Burkina Faso	2339.39(1166.24-3666.11)	4557.59(2262.72-7710.73)	94.82%	44.02(21.96-68.79)	34.14(17.16-56.76)	-0.94 (-0.98 - -0.9)
Burundi	1884.26(931.88-3052.45)	3488.29(1755.29-6242.41)	85.13%	63.64(31.78-103.81)	48.97(25.19-86.04)	-0.68 (-0.84 - -0.52)
Cabo Verde	98.61(48.89-166.22)	180.1(89.07-318.52)	82.64%	48.29(23.91-80.98)	38.07(18.86-67.41)	-0.81 (-0.86 - -0.76)
Cambodia	5821.31(3324.5-8804.84)	11570.27(5983.24-17733.99)	98.76%	105.32(58.21-158)	77.02(40.27-118.5)	-1.4 (-1.57 - -1.23)
Cameroon	3650.64(1885.93-5649.91)	6972.02(3578.6-12362.57)	90.98%	61.83(31.74-96.09)	37.86(19.44-66.54)	-1.62 (-1.84 - -1.4)
Canada	84692.17(45095.45-127203.22)	91857.11(46465.82-140883.76)	8.46%	266.06(142.57-395.54)	174.75(87.86-265.72)	-1.56 (-1.65 - -1.48)
Central African Republic	834.54(414.95-1373.47)	1514.29(760.55-2605.22)	81.45%	54.56(27.55-89.16)	43.85(21.87-75.77)	-0.8 (-0.91 - -0.68)

Chad	2177.14(1070.04-3598.83)	3783.17(1879.25-6604.85)	73.77%	66.03(32.62-109.35)	46.98(23.05-81.36)	-1.23 (-1.46 - -0.99)
Chile	24266.16(13677.66-37154.32)	40812.74(20014.19-66494.53)	68.19%	205.96(117.09-315.04)	177.72(86.66-287.06)	-0.41 (-0.48 - -0.34)
China	1297713.72(775509.73-1968781.52)	1865486.99(1054455.62-2816022.52)	43.75%	130.88(77.46-198.77)	90.75(50.97-135.53)	-0.92 (-1.01 - -0.84)
Colombia	26537.7(14077.23-41269.78)	35694.56(17041.75-60170.66)	34.51%	114.88(60.28-177.95)	63.58(30.33-107.37)	-1.81 (-1.91 - -1.7)
Comoros	154.83(79.19-240.47)	282.66(142.75-469.79)	82.56%	61.29(31.41-96.65)	46.97(23.61-77.49)	-1 (-1.11 - -0.9)
Congo	588.26(298.91-981.99)	2077.59(1048.15-3469.18)	253.18%	43.64(21.95-72.42)	53.95(27.23-92.34)	0.91 (0.78 - 1.03)
Cook Islands	19.77(10.72-29.95)	21.64(11.17-36)	9.46%	134.16(72.29-205.04)	116.51(60.57-196.18)	-0.48 (-0.57 - -0.39)
Costa Rica	2371.2(1237.27-3742.98)	3888.77(1936.17-6487.93)	64.00%	109.74(58.7-174.71)	66.17(32.98-110.35)	-1.43 (-1.62 - -1.23)
Croatia	21864.43(12326.16-32923.72)	19285.79(10529.39-29215.21)	-11.79%	356.76(202.43-529.99)	330.07(184.39-499.03)	0 (-0.08 - 0.08)
Cuba	14217.21(7624.04-22401.92)	19911.19(10225.28-33493.37)	40.05%	136.78(73.67-215.08)	119.44(60.9-199.78)	-0.6 (-0.72 - -0.48)
Cyprus	1949.22(1081.83-2986.91)	3642.85(2017.59-5725.3)	86.89%	242.74(134.57-370.56)	198.15(110.26-311.22)	-0.65 (-0.75 - -0.55)
Czechia	45767.8(25866.49-70173.47)	47324.25(26412.11-73698.29)	3.40%	366.72(207.4-563.35)	304.54(170.73-472.41)	-0.68 (-0.71 - -0.65)
Côte d'Ivoire	3820.9(1814.3-6088.73)	9630.64(4922.96-16473.26)	152.05%	58.89(28.91-96.05)	49.68(25.56-85.54)	-0.5 (-0.7 - -0.3)
Democratic People's Republic of Korea	19452.43(11090.62-28637.54)	30144.32(16654.45-48398.2)	54.96%	99.01(55.66-147.03)	88.84(49.11-142.69)	-0.49 (-0.6 - -0.39)
Democratic Republic of the Congo	8780.3(4369.11-14346.68)	19897.77(10263.66-33414.7)	126.62%	41.66(20.91-68.23)	36.96(19.23-62.22)	-0.16 (-0.31 - -0.01)
Denmark	28456.38(15862.27-41387.75)	20178.22(10451.58-30933.31)	-29.09%	429.2(242.09-623.63)	235.75(120.05-363.56)	-2.32 (-2.48 - -2.16)
Djibouti	165.44(82.55-259.5)	877.88(453.14-1359.31)	430.63%	78.31(39.6-123.34)	90.09(46.04-141.98)	0.68 (0.56 - 0.8)
Dominica	36.51(17.27-65.7)	57.05(28.5-100.83)	56.26%	60.55(29.12-108.62)	68.07(34.05-120.16)	0.42 (0.26 - 0.57)
Dominican Republic	4236.21(2224.51-6761.6)	5571.76(2782.88-9651.31)	31.53%	94.96(48.93-151.09)	52.97(26.64-92.21)	-2.19 (-2.41 - -1.98)
Ecuador	4564.51(2369.33-7216.83)	7385.6(3925.49-12386.12)	61.80%	71.23(37.22-111.16)	42.62(22.65-71.56)	-1.43 (-1.54 - -1.31)
Egypt	38642.62(21488.27-58811.76)	101188.96(58959.33-153117.11)	161.86%	106.44(58.42-161.47)	117.4(67.64-177.99)	0.44 (0.33 - 0.54)

El Salvador	2070.07(999.7-3687.68)	2212.83(1026.92-4424.89)	6.90%	60.33(29.33-107.06)	36.8(17.08-73.5)	-1.61 (-1.7 - -1.51)
Equatorial Guinea	127.66(66.28-201.88)	384.14(206.45-609)	200.91%	51.51(26.42-82.51)	45.98(24.65-74.77)	-0.32 (-0.38 - -0.27)
Eritrea	610.01(314.62-955.97)	1439.65(729.96-2384.14)	136.00%	32.28(16.51-51.03)	30.77(15.61-50.77)	-0.18 (-0.24 - -0.13)
Estonia	4388.02(2434.12-6762.91)	4356.6(2146.67-6795.77)	-0.72%	234.6(128.86-357.96)	227.6(115.15-355.86)	0.24 (0.06 - 0.43)
Eswatini	178.89(90.69-292.3)	263.84(124.75-446.79)	47.49%	47.26(23.86-79.11)	31.64(15.07-54.5)	-1.48 (-1.69 - -1.28)
Ethiopia	8671.49(4270.14-13942.25)	16539.74(8185.45-29980.93)	90.74%	32.37(16.02-51.28)	27.15(13.6-48.64)	-0.33 (-0.56 - -0.11)
Fiji	601.09(310.77-926.57)	788.57(430.21-1303.67)	31.19%	111.76(57.55-171.34)	84.55(46.06-141.98)	-1.08 (-1.18 - -0.98)
Finland	12394.79(7025.42-18335.65)	12394.8(6624.09-18520.49)	0.00%	198.08(112-291.45)	159.68(83.51-236.35)	-0.54 (-0.6 - -0.48)
France	144891.88(77231.99-219200.33)	191203.59(98732.44-296241.8)	31.96%	210.7(114.02-316.64)	207.54(107.1-310.45)	0.05 (-0.01 - 0.11)
Gabon	209.22(101.07-366.47)	781.79(402.41-1332.45)	273.67%	32.96(16.12-56.99)	54.54(27.94-94.14)	2.02 (1.79 - 2.24)
Gambia	396.29(211.19-617.14)	703.54(377.73-1139.67)	77.53%	80.61(42.53-126.46)	50.48(26.84-82.86)	-1.74 (-1.81 - -1.67)
Georgia	7946.11(4420.32-11927.82)	7401.35(4208.03-11315.94)	-6.86%	130.64(73.5-194.6)	152.35(85.59-231.31)	0.72 (0.62 - 0.81)
Germany	302368.88(167732.49-458195.65)	282207.99(150237.16-432766.32)	-6.67%	292.38(164.95-439.54)	229.73(123.15-347.28)	-0.58 (-0.64 - -0.53)
Ghana	3164.32(1520.57-5272.43)	5308.32(2587.28-9210.62)	67.76%	38.95(18.62-64.85)	24.98(12.24-42.83)	-1.58 (-1.65 - -1.52)
Greece	31279.8(17589.47-46971.64)	39087.58(20806.65-58447.48)	24.96%	242.75(139.54-360.27)	242.79(134.13-353.11)	0.13 (0.04 - 0.23)
Greenland	137.12(75.9-214.55)	170.77(95.82-265.24)	24.54%	262.28(147.74-399.76)	240.76(134.77-373.52)	-0.27 (-0.4 - -0.14)
Grenada	41.13(19.63-73.81)	72.55(35.51-134.1)	76.39%	59.22(27.99-105.94)	53.8(25.84-99.27)	-0.29 (-0.39 - -0.19)
Guam	127.6(66.08-203.71)	211.39(113.33-328.57)	65.67%	112.74(59.46-182.43)	110.62(58.26-170.05)	-0.01 (-0.04 - 0.02)
Guatemala	4004.86(2020.26-6710.39)	6271.26(2906.49-11401.45)	56.59%	86.93(43.84-146.7)	49.38(23.03-89.73)	-1.76 (-1.84 - -1.67)
Guinea	2422.62(1199.99-4093.06)	3935.07(1974.04-6803.7)	62.43%	63.86(31.51-107.52)	52.89(26.38-91.96)	-0.68 (-0.8 - -0.56)
Guinea-Bissau	117.91(55.47-213.76)	390.66(195.45-669.88)	231.32%	22.08(10.47-39.04)	32.91(16.6-57.68)	1.65 (1.52 - 1.77)

Guyana	309.46(158.05-492.4)	475.8(247.82-802.93)	53.75%	61.65(31.39-99.94)	60.68(31.61-103.19)	-0.05 (-0.12 - 0.02)
Haiti	2244.19(1138.2-3688.99)	3797.71(1842.06-7407.4)	69.22%	53.5(27.14-88.19)	37.7(18.66-74.89)	-1.34 (-1.48 - -1.2)
Honduras	2243.05(1153.67-3734.48)	3720.5(1827.85-6142.4)	65.87%	86.25(44.49-142.02)	46.86(23.17-77.79)	-2.21 (-2.39 - -2.04)
Hungary	49781.12(28703.93-75615.2)	43120.16(22955.02-66998.23)	-13.38%	382.95(220.68-581.04)	311.73(167.32-481.17)	-0.56 (-0.66 - -0.45)
Iceland	636.22(360.49-988.35)	768.99(403.84-1271.23)	20.87%	242.81(137.93-375.1)	160.83(85.1-266.22)	-1.46 (-1.5 - -1.42)
India	463602.37(248725.83-725155.33)	525137.25(262279.28-854606.35)	13.27%	79.87(43.01-124.69)	40.29(20.49-65.42)	-2.03 (-2.22 - -1.83)
Indonesia	102395.48(58843.26-154355.98)	237330.02(129524.9-352542.68)	131.78%	79.84(45.67-119.34)	79.95(43.09-119.06)	0.13 (0.01 - 0.24)
Iran (Islamic Republic of)	38502.12(19761.89-61584.44)	100890.89(51496.87-166521.82)	162.04%	113.29(57.91-177.13)	101.02(51.92-168.57)	-0.25 (-0.32 - -0.18)
Iraq	13093.27(7073.74-20121.61)	38797.03(21971.87-60335.52)	196.31%	131.64(69.43-201.72)	120.63(68.77-187.8)	-0.17 (-0.26 - -0.09)
Ireland	10128.57(5394.99-15151.4)	11163.76(5940.69-17368.07)	10.22%	269.68(144.53-404.59)	163.17(85.94-254.13)	-1.73 (-1.86 - -1.59)
Israel	10359.94(5816.41-16230.16)	16486.91(8485.9-26358.14)	59.14%	223.62(126.92-350.06)	162.84(84.76-259.76)	-1.03 (-1.08 - -0.97)
Italy	172472.8(92799.95-259659.86)	157459.1(85059.59-248995.78)	-8.70%	238.5(127.25-354.99)	172.68(92.69-271.6)	-1.03 (-1.1 - -0.95)
Jamaica	1544.06(812.34-2417.24)	1733.34(844.64-3032.61)	12.26%	90.3(47.49-140.03)	56.7(27.57-99.18)	-1.79 (-2 - -1.59)
Japan	385884.89(216721.42-573183.03)	324532.03(174884-526226.36)	-15.90%	242.79(136.88-357.7)	174.21(93.51-280.7)	-1.03 (-1.13 - -0.92)
Jordan	3102.29(1750.3-4674.54)	18190.8(10413.14-27453.37)	486.37%	161.79(89.34-246.98)	169.16(98.09-258.44)	0.18 (0.12 - 0.24)
Kazakhstan	19261.3(10918.58-29682.01)	26555.26(13289.23-40970.04)	37.87%	128.31(72.01-196.59)	124.18(62.11-191.52)	0.01 (-0.19 - 0.21)
Kenya	6845.03(3490.79-10800.89)	16400.75(8872.87-26303.36)	139.60%	65.65(33.03-104.79)	51.92(27.64-84.18)	-0.94 (-1.03 - -0.86)
Kiribati	73.32(40.88-112.09)	158.46(88.3-243.97)	116.12%	153.44(86.39-233.99)	162.57(90.87-247.41)	0.07 (-0.17 - 0.31)
Kuwait	1693.81(937.77-2643.44)	6136.23(3321.65-9584.48)	262.27%	131.85(71.58-198.47)	112.02(60.15-177.84)	-0.61 (-0.65 - -0.57)
Kyrgyzstan	4113.49(2352.76-6242.06)	8275.01(4481.33-12366.33)	101.17%	125.63(71.01-191.16)	129.66(69.57-194.19)	0.47 (0.3 - 0.65)
Lao People's Democratic Republic	2466.52(1328.23-3777.7)	5278.16(3001.48-8238.61)	113.99%	97.85(52.54-149)	86.33(49.51-134.17)	-0.29 (-0.37 - -0.21)

Latvia	7128.51(4000.77-10797.39)	5465.2(3179.87-8306.89)	-23.33%	216.39(120.93-324.35)	196.35(110.47-296.47)	-0.05 (-0.18 - 0.07)
Lebanon	3576.72(1875.96-5498.11)	11224.94(6231.1-17445.79)	213.83%	141.66(73.7-218.11)	189.59(104.86-295.38)	1.14 (0.85 - 1.42)
Lesotho	567.42(290.96-906.57)	719.98(393.58-1107.88)	26.89%	60.33(30.87-97.48)	50.4(27.53-78.09)	-0.68 (-0.8 - -0.57)
Liberia	705.43(357.65-1134.02)	1357.19(666.99-2339.26)	92.39%	50.43(25.53-79.45)	38.51(19.07-67.01)	-0.95 (-1.1 - -0.81)
Libya	2607.58(1424.08-4016.71)	8421.59(4750.65-12784.69)	222.97%	105.52(56.45-162.71)	111.71(62.75-169.98)	0.43 (0.29 - 0.57)
Lithuania	8174.33(4620.57-12577.38)	8671.24(4566.15-13206.21)	6.08%	196.45(111.45-300.79)	215.55(115.57-321.45)	0.59 (0.5 - 0.68)
Luxembourg	1150.42(633.11-1783.38)	1686.07(915.4-2688.64)	46.56%	236.47(130.91-365.71)	191.59(104.68-301.08)	-0.73 (-0.8 - -0.66)
Madagascar	5199.38(2809.93-8040.23)	8959.21(5011.98-14128.7)	72.31%	79.98(42.91-122.6)	51.06(27.78-80.26)	-1.51 (-1.77 - -1.25)
Malawi	3283.97(1695.38-5132.49)	6054.71(3159.73-9768.69)	84.37%	70.35(36.33-112.04)	61.83(32.27-100.37)	-0.45 (-0.49 - -0.4)
Malaysia	9223.69(5047.55-13726.76)	20146.25(10975.59-31347.58)	118.42%	74.86(40.67-110)	57.72(31.59-91.07)	-1.02 (-1.15 - -0.89)
Maldives	111.13(63-165.34)	426.63(233.09-688.11)	283.90%	98.82(55.39-146.53)	82.44(45.35-132.37)	-0.73 (-0.87 - -0.58)
Mali	1624.68(802.74-2635.7)	5320.4(2627.37-9043.47)	227.47%	32.07(15.8-52.22)	44.39(21.94-74.88)	1.3 (1.15 - 1.45)
Malta	1115.91(621.42-1687.59)	1398.94(748.65-2214.76)	25.36%	258.29(144.57-389.29)	186.88(101.49-294.89)	-1 (-1.12 - -0.88)
Marshall Islands	20.01(10.09-32.55)	29.46(15.78-47.92)	47.23%	83.55(42.05-132.36)	79.29(41.79-129.46)	-0.18 (-0.2 - -0.17)
Mauritania	653.32(332.69-1041.65)	1097.01(551.9-1837.8)	67.91%	52.28(26.94-83.56)	39.17(19.88-65.85)	-1.12 (-1.23 - -1.01)
Mauritius	747.65(401.1-1136.25)	1092.56(601.08-1755.03)	46.13%	81.92(43.73-122.69)	63.8(35.05-103.42)	-0.67 (-0.78 - -0.55)
Mexico	57086.14(31144.36-89128.19)	92161.54(46514.27-143066.68)	61.44%	105.16(58.05-163.88)	66.53(33.47-103.05)	-1.65 (-1.79 - -1.5)
Micronesia (Federated States of)	87.36(47.11-132.64)	134.19(75.64-214.45)	53.61%	143.35(79.27-221.44)	141.39(79.43-223.96)	0.01 (-0.07 - 0.09)
Monaco	107.75(54.31-171.83)	116.11(60.93-193.53)	7.76%	234.43(118.75-376.37)	193.62(105.34-325.29)	-0.67 (-0.7 - -0.64)
Mongolia	1813.92(994.15-2788.28)	4621.72(2475.8-7042.07)	154.79%	143.48(76.63-222.4)	145.11(77.57-221.43)	0.11 (0.08 - 0.15)
Montenegro	2367.81(1333.84-	3249.66(1807.76-	37.24%	363.97(203.19-	368.35(205.63-	0.14 (0.04 - 0.25)

	3679.48)	4961.21)		565.06)	547.87)	
Morocco	18185.3(9813.41-27418.71)	30389.49(16830.56-49263.81)	67.11%	103.31(54.82-157.91)	77.15(42.73-125.2)	-1.01 (-1.08 - -0.95)
Mozambique	3866.96(1936.94-6319.02)	8346.34(4283.57-13887.39)	115.84%	49.41(24.7-81)	53.34(27.67-86.41)	0.51 (0.13 - 0.89)
Myanmar	27479.1(15162.15-41085.73)	30811.32(16090.05-48120.7)	12.13%	101.19(55.45-151.28)	55.92(29.17-87.1)	-2.19 (-2.32 - -2.07)
Namibia	718.84(355.59-1163.42)	1293.88(656.06-2170.83)	80.00%	91.77(45.17-149.12)	67.11(34.36-112.63)	-1.18 (-1.28 - -1.08)
Nauru	9.69(5.27-14.89)	12.78(7.08-19.34)	31.89%	154.11(84.93-239.44)	147.79(79.95-224.28)	-0.1 (-0.17 - -0.03)
Nepal	30939.59(17456.21-46878.53)	40623.49(23071.59-64459.44)	31.30%	266.77(149.09-405.76)	151.74(86.31-241.36)	-2.06 (-2.3 - -1.82)
Netherlands	45734.45(25706.46-67350.25)	45754.52(23313.85-70051.37)	0.04%	255.6(144.37-375.03)	178.72(90.46-269.91)	-1.09 (-1.17 - -1)
New Zealand	10436.57(5509.02-16009.05)	11310.11(5610.77-17390.81)	8.37%	281.36(149.16-428.28)	172.58(85.61-262.59)	-1.61 (-1.67 - -1.55)
Nicaragua	1656.33(810.98-2945.35)	3337.92(1616.03-5802.65)	101.53%	79.1(39.2-139.26)	55.71(27.22-96.91)	-0.83 (-0.94 - -0.72)
Niger	1089.76(527.59-1836.56)	4100.81(1839.13-7402.23)	276.30%	26.15(12.58-44.44)	35.68(16.48-63.81)	1.06 (1.01 - 1.11)
Nigeria	21556.99(10187.73-38448.37)	27812.92(12735.85-50977.45)	29.02%	40.64(19.02-72.34)	21.23(9.82-38.73)	-2.15 (-2.31 - -1.99)
Niue	1.9(0.98-3.03)	1.74(0.91-2.94)	-8.42%	93.91(48.38-149.5)	86.68(44.48-146.36)	-0.34 (-0.42 - -0.26)
North Macedonia	6371.08(3535.03-9905.43)	8419.92(4567.28-12890.39)	32.16%	307.29(170.96-475.48)	321.92(180-485.59)	0.23 (0.19 - 0.27)
Northern Mariana Islands	44.26(22.08-73.05)	61.64(32.76-102.01)	39.27%	117.68(60.64-194.44)	101.6(54.19-169.63)	-0.54 (-0.59 - -0.49)
Norway	12226.88(7161.66-19043.46)	11509.21(5737.4-18730.07)	-5.87%	239.61(141.51-373.67)	157.74(77.77-255.24)	-1.63 (-1.74 - -1.52)
Oman	1351.87(710.84-2259.76)	3296.14(1700.04-5453.65)	143.82%	102.56(53.1-164.34)	67.27(34.76-111.57)	-1.26 (-1.52 - -1.01)
Pakistan	64285.03(34960.76-101995.11)	115323.39(62105.9-199095.47)	79.39%	102.43(55.55-161.71)	76.17(41.06-128.05)	-0.93 (-1.01 - -0.84)
Palau	11.65(5.84-18.65)	21.29(11.03-35.42)	82.75%	93.99(47.27-150.02)	87.65(45.42-146.23)	-0.35 (-0.42 - -0.28)
Palestine	1285.83(687.2-1964.8)	4456.89(2450.07-6823.45)	246.62%	123.77(65.82-193.14)	123.06(67.57-188.86)	-0.16 (-0.22 - -0.11)
Panama	1342.98(637.83-	1422.77(639.05-	5.94%	76.79(36.44-129.8)	33.33(14.98-57.27)	-3.15 (-3.31 - -

	2287.79)	2451.51)				2.98)
Papua New Guinea	2754.68(1458.56-4241.88)	7630.72(4002.68-11877.78)	177.01%	106.53(54.31-165.83)	97.11(50.78-154.71)	-0.39 (-0.47 - -0.32)
Paraguay	3855.4(2009.12-5926.56)	6763.9(3606.29-11423.27)	75.44%	147.14(76.43-224.39)	113(60.41-191.16)	-1 (-1.09 - -0.9)
Peru	7076.96(3616.31-11987.04)	15286.78(7806.18-25317.81)	116.01%	48.87(25.08-81.76)	41.24(21.07-68.7)	-0.79 (-0.9 - -0.68)
Philippines	48634.5(27215.08-73307.41)	76803.28(39700.93-125344.24)	57.92%	127.55(72.29-192.28)	79.27(40.7-127.94)	-1.61 (-1.78 - -1.44)
Poland	172926.7(96929.01-254946.33)	169266.4(86976.12-253248.7)	-2.12%	389.77(220.31-568.92)	299.76(153.3-448.72)	-0.83 (-0.89 - -0.77)
Portugal	24202.85(12760.92-36996.51)	28918.21(15261.39-43917.11)	19.48%	203.62(107.52-315.23)	193.46(102.14-290.73)	-0.02 (-0.08 - 0.05)
Puerto Rico	2912.76(1431.86-4814.84)	3811.33(1801.44-6222.84)	30.85%	80.62(39.58-134.14)	72.93(35.53-117.15)	-0.36 (-0.39 - -0.33)
Qatar	418.1(227.8-659.53)	3580.85(1860.51-5805.75)	756.46%	102.86(53.44-164.25)	97.29(51.13-158.32)	0.02 (-0.07 - 0.11)
Republic of Korea	72180.4(42033.83-109685.2)	110600.74(58026.82-179107.37)	53.23%	175.17(100.41-267.5)	142.17(74.46-227.5)	-0.71 (-0.76 - -0.66)
Republic of Moldova	6886.44(3833.78-10845.75)	8884.4(4894.73-13748.97)	29.01%	146.7(82.16-230.04)	168.37(92.12-257.84)	0.51 (0.47 - 0.55)
Romania	80745.63(44543.48-125509.71)	75951.85(41380.81-115937.44)	-5.94%	299.15(164.7-462.31)	275.24(153.55-417.81)	-0.46 (-0.54 - -0.38)
Russian Federation	309405.01(167568.36-461280.46)	455074.44(239283.48-683666.28)	47.08%	174.97(94.28-258.07)	211.97(113.61-317.37)	0.91 (0.75 - 1.06)
Rwanda	3443.57(1708.01-5544.18)	7628.4(3793.78-12817.18)	121.53%	98.86(49.47-158.45)	96.61(47.95-166.06)	-0.01 (-0.19 - 0.17)
Saint Kitts and Nevis	15.57(7.38-28.88)	21.67(10.04-41.45)	39.18%	48.54(22.99-88.8)	36.18(16.8-70)	-1.21 (-1.35 - -1.07)
Saint Lucia	70.72(35.1-120.79)	144.94(75.19-269.32)	104.95%	76.02(37.63-130.51)	63.77(33-118.77)	-0.9 (-1.1 - -0.69)
Saint Vincent and the Grenadines	42.77(21.77-71.51)	71.61(36.44-130.36)	67.43%	58.64(29.91-97.55)	51.54(25.73-93.66)	-0.45 (-0.48 - -0.42)
Samoa	142.21(73.31-219.36)	224.6(123.25-351.62)	57.94%	140.35(72.39-216.64)	129.42(70.98-203.79)	-0.53 (-0.65 - -0.4)
San Marino	59.12(32.73-93.95)	94.65(50.39-161.51)	60.10%	228.67(126.83-364.87)	173.98(94.38-296.39)	-1.04 (-1.1 - -0.99)
Sao Tome and Principe	17.06(8.36-29.9)	44.71(21.65-79.61)	162.08%	24.8(12.13-43.09)	28.77(13.86-52.14)	0.53 (0.25 - 0.8)
Saudi Arabia	6302.8(3177.31-10228.23)	28544.18(14698.59-44718.38)	352.88%	61.44(30.99-101.8)	84.24(44.07-133.17)	0.85 (0.7 - 1.01)

Senegal	2467.42(1338.84-3847.77)	3685.19(1808.01-5912.62)	49.35%	60.24(32.54-93.44)	34.32(17.03-55.15)	-2.07 (-2.25 - -1.9)
Serbia	38800.19(22193.99-57739.56)	44664.08(23431.96-67767.93)	15.11%	350.66(200.26-522.97)	349.78(183.69-527.78)	0.03 (-0.06 - 0.12)
Seychelles	44.67(23.37-71.04)	89.71(48.02-148.27)	100.83%	78.16(40.8-123.51)	70.04(36.99-115)	-0.53 (-0.6 - -0.47)
Sierra Leone	2228.67(1185.79-3396.52)	2772.95(1538.12-4450.06)	24.42%	85.68(45.04-130.27)	56.41(31.22-91.43)	-1.37 (-1.43 - -1.31)
Singapore	2713.59(1378.58-4383.39)	6306.78(2978.55-10555.96)	132.41%	87.25(44.14-140.2)	73.59(34.75-124.2)	-0.18 (-0.29 - -0.06)
Slovakia	15941.48(8739.49-24528.57)	20516.54(11116.37-31933.35)	28.70%	277.47(153.21-422.91)	264.49(145.39-412.6)	-0.01 (-0.06 - 0.04)
Slovenia	6230.77(3331.95-9762.48)	8442.16(4423.86-13294.26)	35.49%	265.58(142.23-412.64)	274.31(146.04-421.68)	0.39 (0.28 - 0.51)
Solomon Islands	232.74(127.8-364)	793.41(433.19-1262.24)	240.90%	125.74(69.16-191.89)	135.42(73.01-217.07)	0.46 (0.35 - 0.56)
Somalia	2191.24(1085.07-3724.51)	4937.93(2485.32-8420.75)	125.35%	53.17(26.65-91.96)	46.89(23.87-78.49)	-0.42 (-0.43 - -0.41)
South Africa	32500.27(18182.07-49368.31)	46748.79(24817.95-72340.33)	43.84%	130.76(71.96-201.06)	78.34(41.03-121.86)	-1.72 (-1.97 - -1.47)
South Sudan	942.47(447.64-1588.34)	2108.63(1038.76-3599)	123.73%	32.61(15.81-55.02)	37.16(18.39-63.87)	0.66 (0.57 - 0.75)
Spain	88328.91(49498.02-129044.75)	111501.57(57539.21-170912.95)	26.23%	199.51(111.97-288.91)	160.6(82.97-242.21)	-0.34 (-0.47 - -0.2)
Sri Lanka	9457.7(5282.52-14661.83)	9619.5(4729.87-15126.56)	1.71%	71.19(38.82-108.7)	36.92(18.16-57.44)	-2.12 (-2.17 - -2.08)
Sudan	6222.11(3080.71-10236.7)	15605(7890.81-26915.82)	150.80%	52.54(26.18-84.63)	55.01(27.92-94.79)	0.13 (-0.03 - 0.29)
Suriname	309.47(169.07-498.27)	545.96(286.04-942.11)	76.42%	104.41(57.08-167.71)	82.75(43.22-142.58)	-0.89 (-1.04 - -0.73)
Sweden	16104.78(9090.63-25982.68)	22381.6(11259.73-35350.82)	38.97%	137.26(78.62-213.46)	147.96(73.83-236.3)	0.04 (-0.15 - 0.24)
Switzerland	24517.45(13744.72-35690.63)	29131.02(15222.07-45445.15)	18.82%	285.43(160.73-414.34)	227(120.69-349.47)	-0.63 (-0.75 - -0.51)
Syrian Arab Republic	9546.84(4937.44-14900.08)	20374.72(10815.79-31167.16)	113.42%	142.21(73.4-218.71)	118.59(62.44-182.58)	-0.63 (-0.73 - -0.53)
Taiwan	23064.82(12376.45-33929.57)	41612.61(22053.62-63596.86)	80.42%	123.11(65.95-183.16)	119.29(64.05-181.8)	-0.04 (-0.18 - 0.1)
Tajikistan	3605.48(1919.91-5638.18)	5431.07(2803.95-9518.38)	50.63%	112.97(60.73-174.24)	67.79(34.85-119.08)	-1.67 (-1.74 - -1.59)
Thailand	35625.41(20478.54-53661.89)	55742.2(29891.59-86368.84)	56.47%	80.35(44.82-122.32)	56.69(30.29-86.99)	-1.07 (-1.22 - -0.92)

Timor-Leste	365.56(202.25-550.9)	673.51(381.99-1007.7)	84.24%	83.16(45.48-128.68)	70.04(39.41-104.99)	-0.6 (-0.63 - -0.58)
Togo	1079.94(538.38-1753.42)	2129.95(1025.46-3853.45)	97.23%	63.98(31.74-104.1)	38.84(18.78-69.99)	-1.83 (-2.19 - -1.47)
Tokelau	1.93(1.05-3.02)	2.43(1.39-3.89)	25.91%	147(81.04-231.1)	149.95(85.68-239.46)	0.14 (0.08 - 0.2)
Tonga	81.69(44.53-129.32)	90.98(49.03-142.54)	11.37%	131.43(70.96-207.53)	104.77(56.33-164.35)	-0.85 (-0.9 - -0.81)
Trinidad and Tobago	834.2(442.75-1376.25)	1500.6(784.83-2573.33)	79.88%	86.61(45.93-142.15)	82.28(42.71-141.21)	-0.05 (-0.09 - -0.01)
Tunisia	7359.79(3996.12-10903.49)	15958.13(8915.07-24538.98)	116.83%	126.02(68.15-188.16)	114.03(63.74-175.76)	-0.31 (-0.36 - -0.26)
Turkmenistan	3147.62(1709.01-4847.26)	5201.36(2852.67-8598.25)	65.25%	132.48(72.83-201.88)	99.74(54.98-164.86)	-1.04 (-1.23 - -0.85)
Tuvalu	8.32(4.08-13.22)	10.41(5.69-16.37)	25.12%	105.84(52.73-168.02)	103.95(57.53-164.62)	-0.05 (-0.11 - 0)
Türkiye	87115.59(49185.1-127588.46)	165497.52(91174.38-254759.75)	89.97%	200.61(113.15-295.4)	168.62(93.18-258.76)	-0.54 (-0.64 - -0.45)
Uganda	4004.82(2037.62-6549.7)	9898.65(4982.76-16123.29)	147.17%	50.16(25.42-82.06)	49(23.8-80.31)	-0.25 (-0.5 - 0.01)
Ukraine	132005.45(75147.21-201058.99)	119848.76(62323.52-180934.15)	-9.21%	206.16(116.67-316)	194(100.6-292.78)	-0.03 (-0.24 - 0.18)
United Arab Emirates	1514.5(737.9-2386.82)	9709.62(4989.17-16057.16)	541.11%	87.68(42.17-137.89)	60.21(31.34-103.02)	-1.28 (-1.46 - -1.1)
United Kingdom	192241.81(104980.38-281028.66)	182614.44(96843.97-275922.71)	-5.01%	271.98(149.27-392.46)	193.18(107.36-287.72)	-0.97 (-1.06 - -0.87)
United Republic of Tanzania	13501.73(7212.17-20945.56)	31867.59(17096.08-50462.75)	136.03%	99.53(53.66-152.61)	87.94(47.08-138.4)	-0.53 (-0.61 - -0.45)
United States Virgin Islands	65.51(32.88-109)	62.66(31.11-101.29)	-4.35%	62.03(31.24-101.78)	45.91(22.58-74.66)	-1.01 (-1.21 - -0.82)
United States of America	801191.01(430182.11-1169862.43)	856993.14(420048.99-1293736.78)	6.96%	280.92(151.62-407.82)	196.16(95.94-290.47)	-0.97 (-1.03 - -0.91)
Uruguay	6412.33(3460.61-10028.86)	7987.97(4356.21-12160.7)	24.57%	191.56(104.37-301.41)	193.23(104.31-294.9)	-0.11 (-0.22 - -0.01)
Uzbekistan	11861.09(6357.79-18122.58)	29693.73(15793.46-49049.17)	150.35%	90.95(48.14-140.3)	88.47(46.66-144.52)	0.07 (-0.14 - 0.28)
Vanuatu	80.66(41.29-127.03)	163.71(83.98-271.01)	102.96%	92.43(47.31-142.07)	69.82(35.97-116.09)	-1.2 (-1.31 - -1.08)
Venezuela (Bolivarian Republic of)	14399.03(7222.8-24198.75)	19636.58(8767.21-34318.43)	36.37%	112.28(57.48-188.25)	61.52(27.4-107.57)	-1.95 (-2.09 - -1.81)
Viet Nam	40435.18(23258.23-61069.4)	89544.98(47959.08-132577.83)	121.45%	91.52(52.28-137.77)	77.86(41.8-115.39)	-0.55 (-0.6 - -0.51)
Yemen	9781.46(5151.22-	24786.5(12165.11-	153.40%	142.21(74.87-217.99)	113.96(55.76-188.18)	-0.92 (-1.02 - -

	15318.26)	40371.49)				0.82)
Zambia	2406.69(1180.83-3778.56)	4359.29(2338.76-7125.29)	81.13%	70.79(34.44-113.59)	48.48(24.9-78.66)	-1.68 (-1.85 - -1.5)
Zimbabwe	5078.69(2655.48-7684.59)	6611.83(3392.2-10307.07)	30.19%	101.01(52.47-154.19)	67.97(35.42-105.14)	-1.35 (-1.4 - -1.3)

Note: DALY = disability-adjusted life year; ASDR = age-standardized DALY rate per 100,000 population; SDI = Socio-demographic Index; GBD = Global Burden of Disease; UI = uncertainty interval; CI = confidence interval; EAPC = estimated annual percentage change. Values are for both sexes combined.

Supplementary Figures S1-S9

Supplementary Figure S1. Geographic distribution of age-standardized DALY rates and EAPC for smoking-attributable musculoskeletal disorders in a secondary dataset analysis of GBD 2021 estimates, 1990-2021

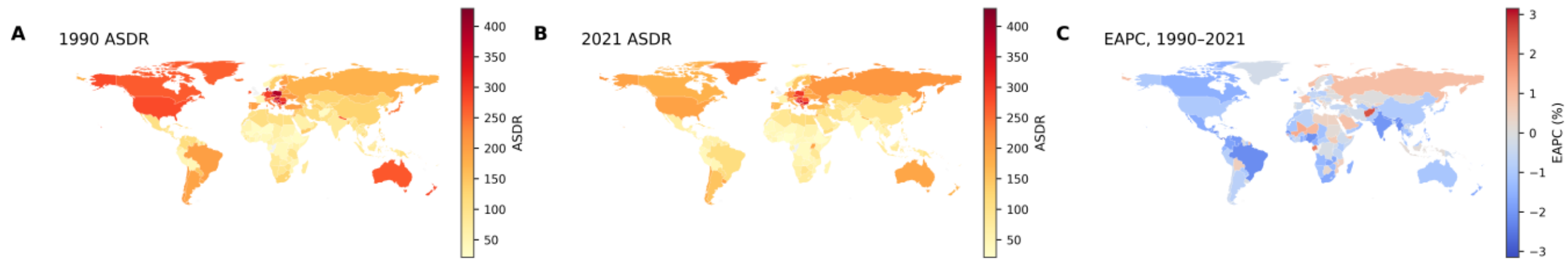

Note: Panel A shows 1990 ASDR, Panel B shows 2021 ASDR, and Panel C shows EAPC in ASDR from 1990 to 2021. ASDR = age-standardized DALY rate per 100,000 population; DALY = disability-adjusted life year; EAPC = estimated annual percentage change; GBD = Global Burden of Disease.

Supplementary Figure S2. Country-age heat map of DALY rates for smoking-attributable musculoskeletal disorders across 204 countries and territories in a secondary dataset analysis of GBD 2021 estimates, 2021

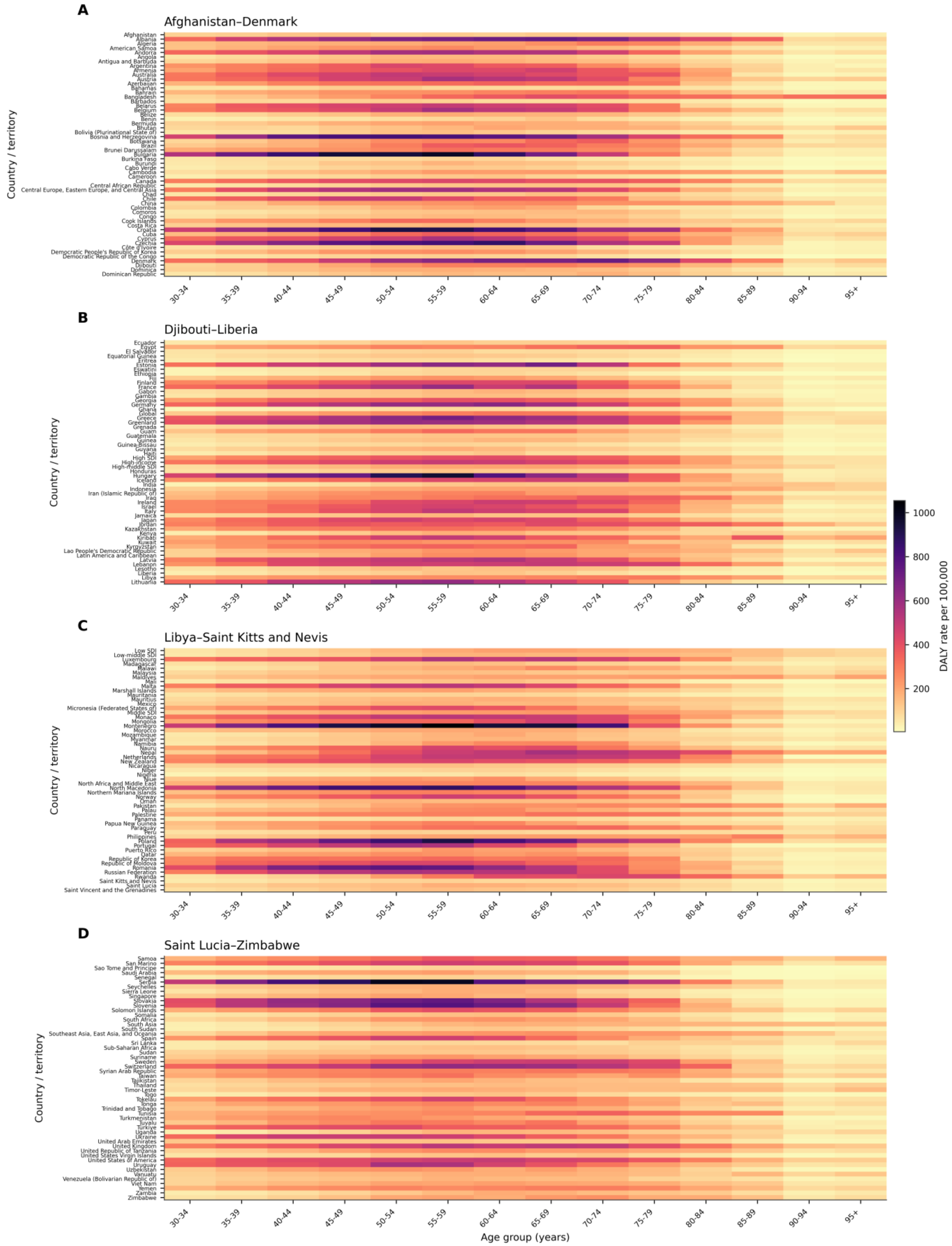
A
Afghanistan–Denmark
B
Djibouti–Liberia
C
Libya–Saint Kitts and Nevis
D
Saint Lucia–Zimbabwe
Country / territory
30-34
35-39
40-44
45-49
50-54
55-59
60-64
65-69
70-74
75-79
80-84
85-89
90-94
95+
Age group (years)
DALY rate per 100,000
200
400
600
800
1000

Note: Heat maps display DALY rates per 100,000 population across age groups. Panels divide countries alphabetically for readability. DALY = disability-adjusted life year; GBD = Global Burden of Disease.

Supplementary Figure S3. Complete joinpoint regression panels for global and SDI-specific ASDRs for smoking-attributable musculoskeletal disorders in a secondary dataset analysis of GBD 2021 estimates, 1990-2021

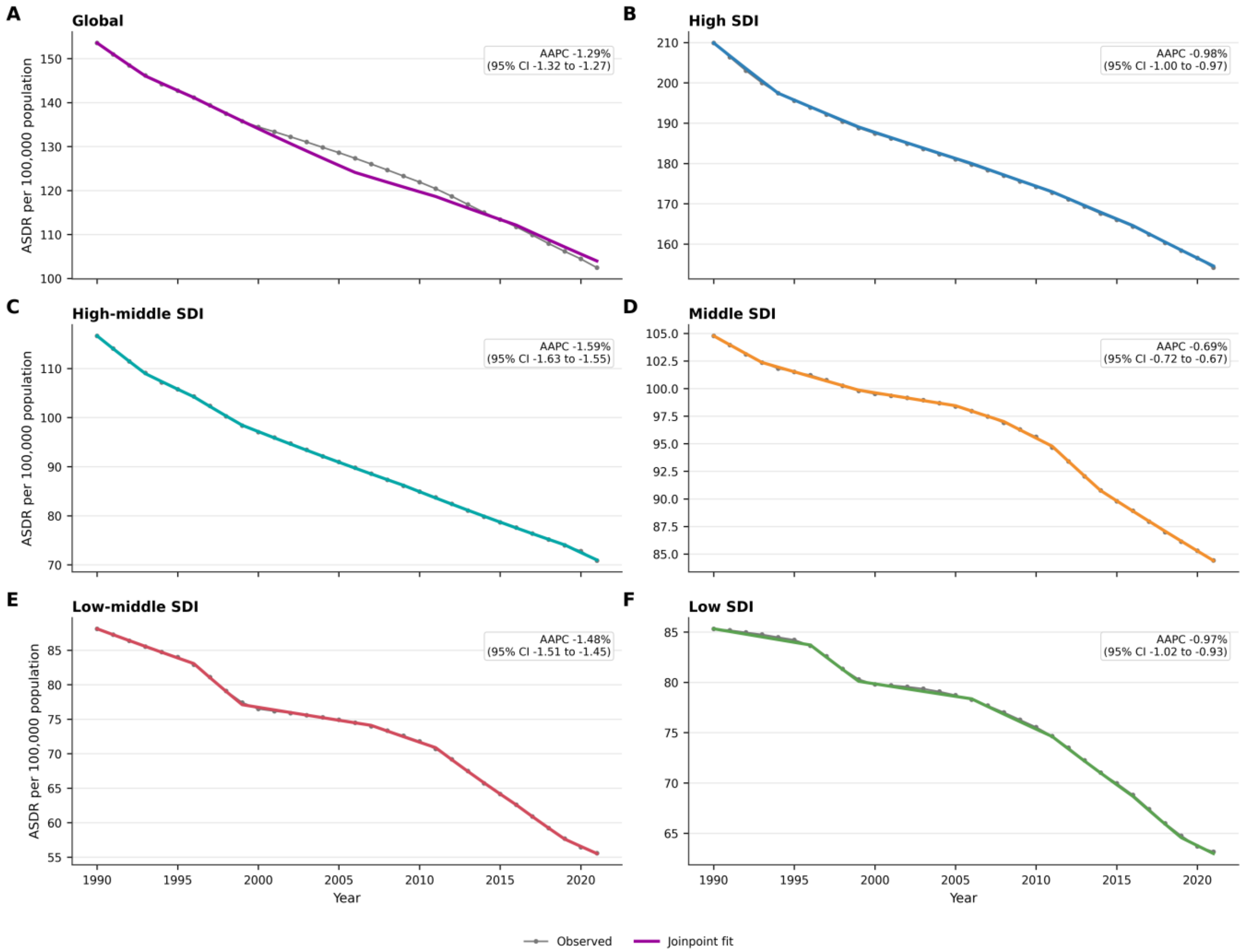

Note: Panels show observed ASDRs and joinpoint-fitted trends. ASDR = age-standardized DALY rate per 100,000 population; SDI = Socio-demographic Index; GBD = Global Burden of Disease.

Supplementary Figure S4. SDI-stratified burden indicators for smoking-attributable musculoskeletal disorders in a secondary dataset analysis of GBD 2021 estimates, 1990 and 2021

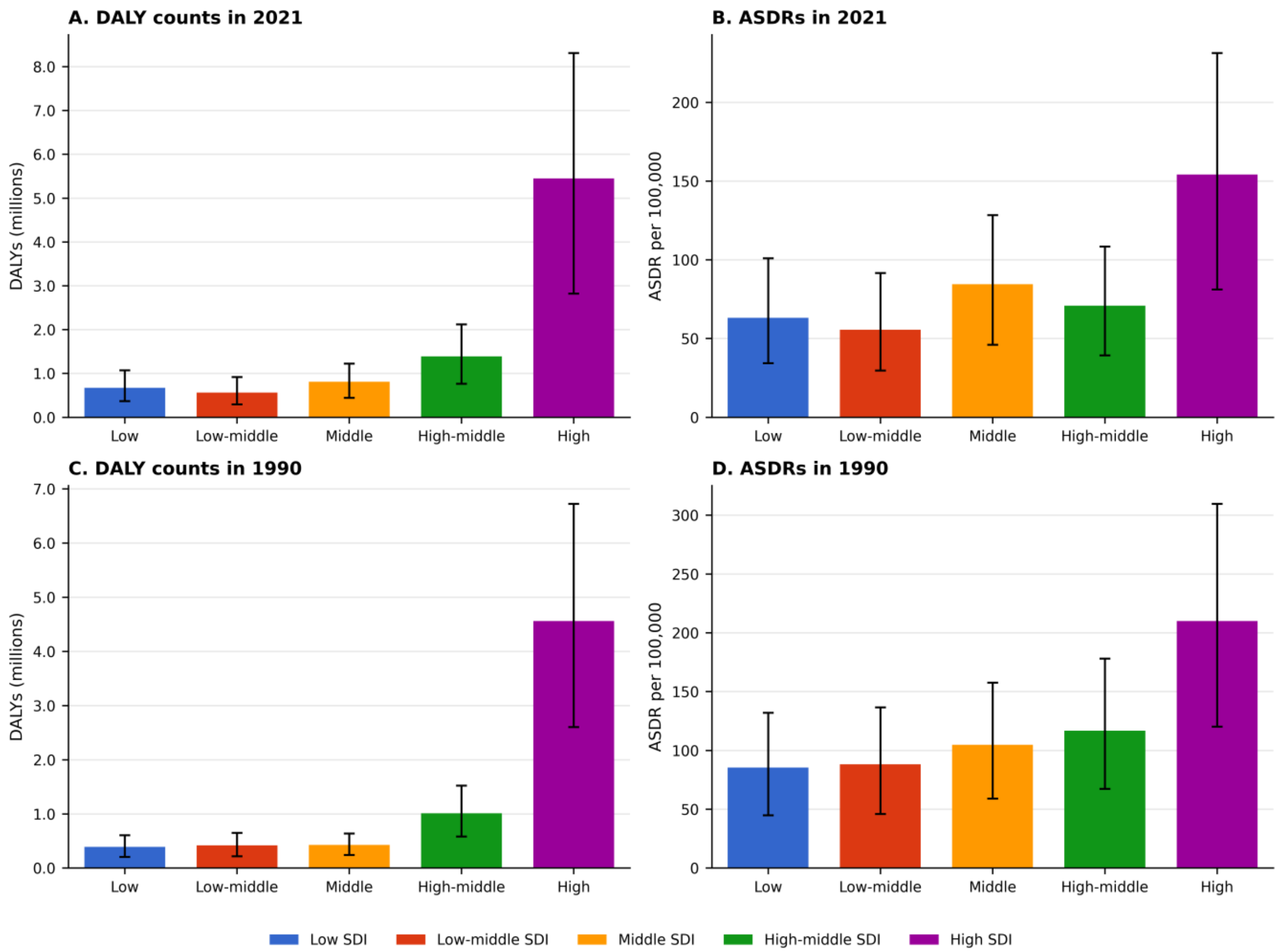

Note: Panels show DALY counts and ASDRs with 95% uncertainty intervals. DALY = disability-adjusted life year; ASDR = age-standardized DALY rate per 100,000 population; SDI = Socio-demographic Index; GBD = Global Burden of Disease; UI = uncertainty interval.

Supplementary Figure S5. Temporal trends in smoking-attributable musculoskeletal disorder burden by SDI quintile in a secondary dataset analysis of GBD 2021 estimates, 1990-2021

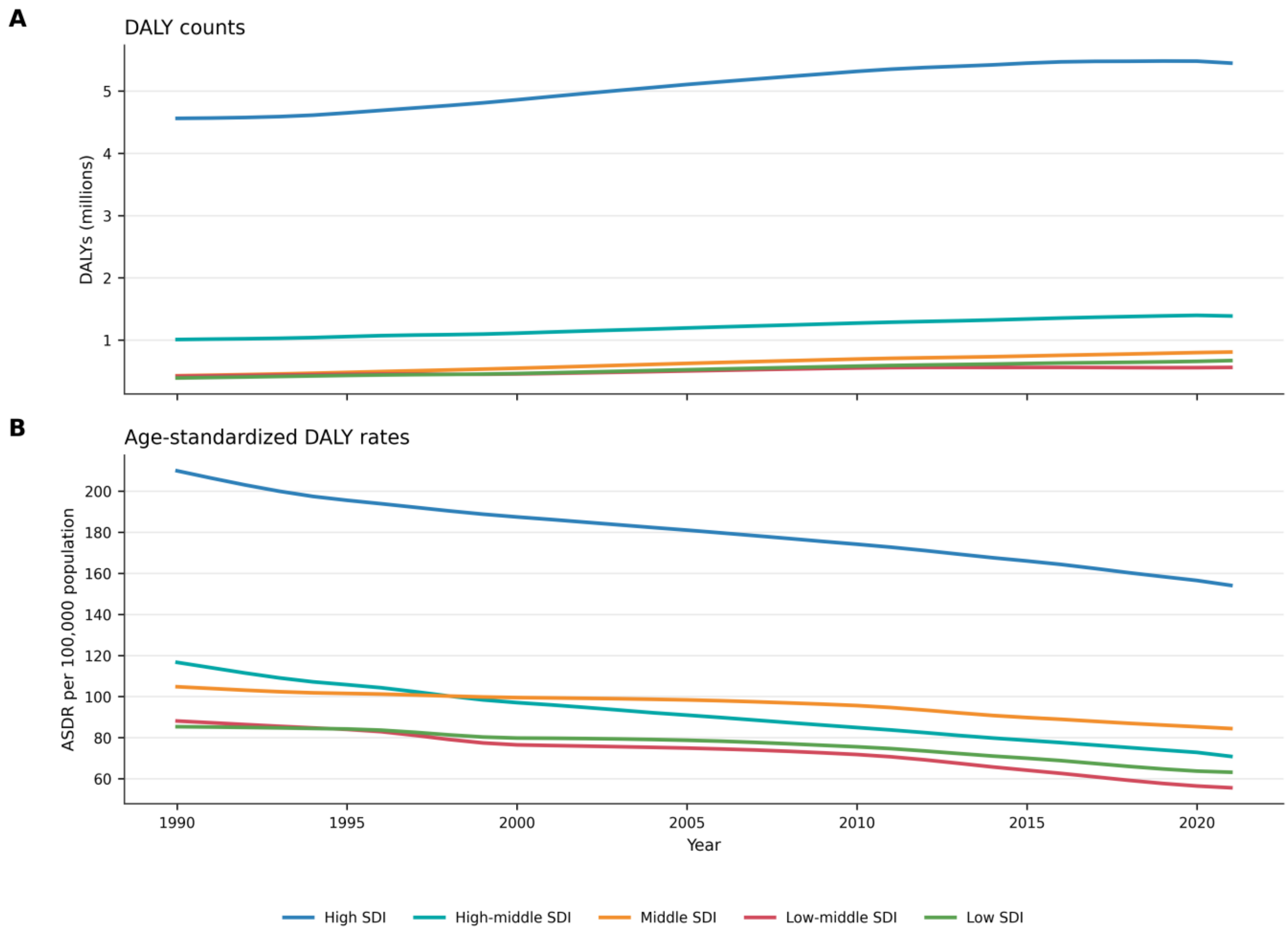

Note: Panel A shows DALY counts in millions. Panel B shows ASDRs per 100,000 population. DALY = disability-adjusted life year; ASDR = age-standardized DALY rate; SDI = Socio-demographic Index; GBD = Global Burden of Disease.

Supplementary Figure S6. Frontier analysis of smoking-attributable musculoskeletal disorder burden across countries and territories in a secondary dataset analysis of GBD 2021 estimates, 1990-2021

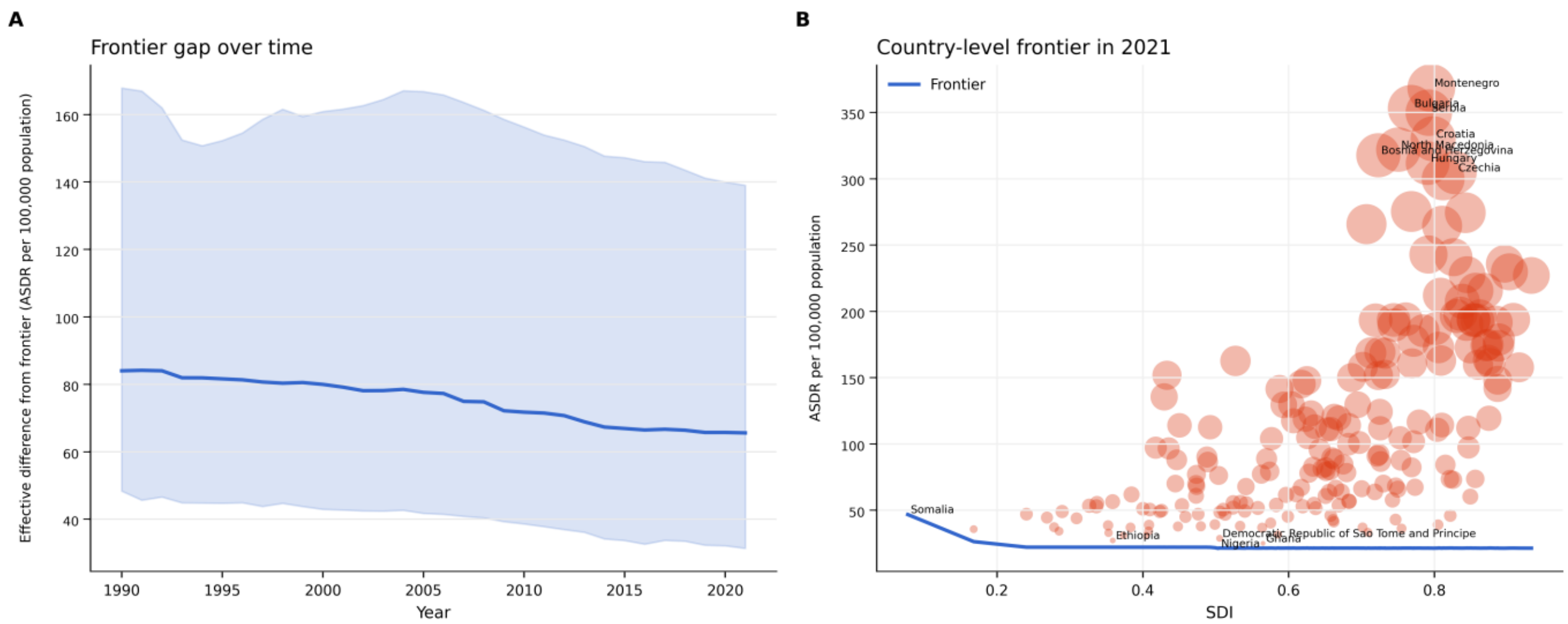

Note: Panel A shows the median effective difference from the frontier, expressed as ASDR per 100,000 population, with interquartile range. Panel B shows the 2021 frontier curve and country-level ASDRs per 100,000 population. ASDR = age-standardized DALY rate; DALY = disability-adjusted life year; GBD = Global Burden of Disease.

Supplementary Figure S7. SDI-related health inequality in smoking-attributable musculoskeletal disorder burden in a secondary dataset analysis of GBD 2021 estimates, 1990 and 2021

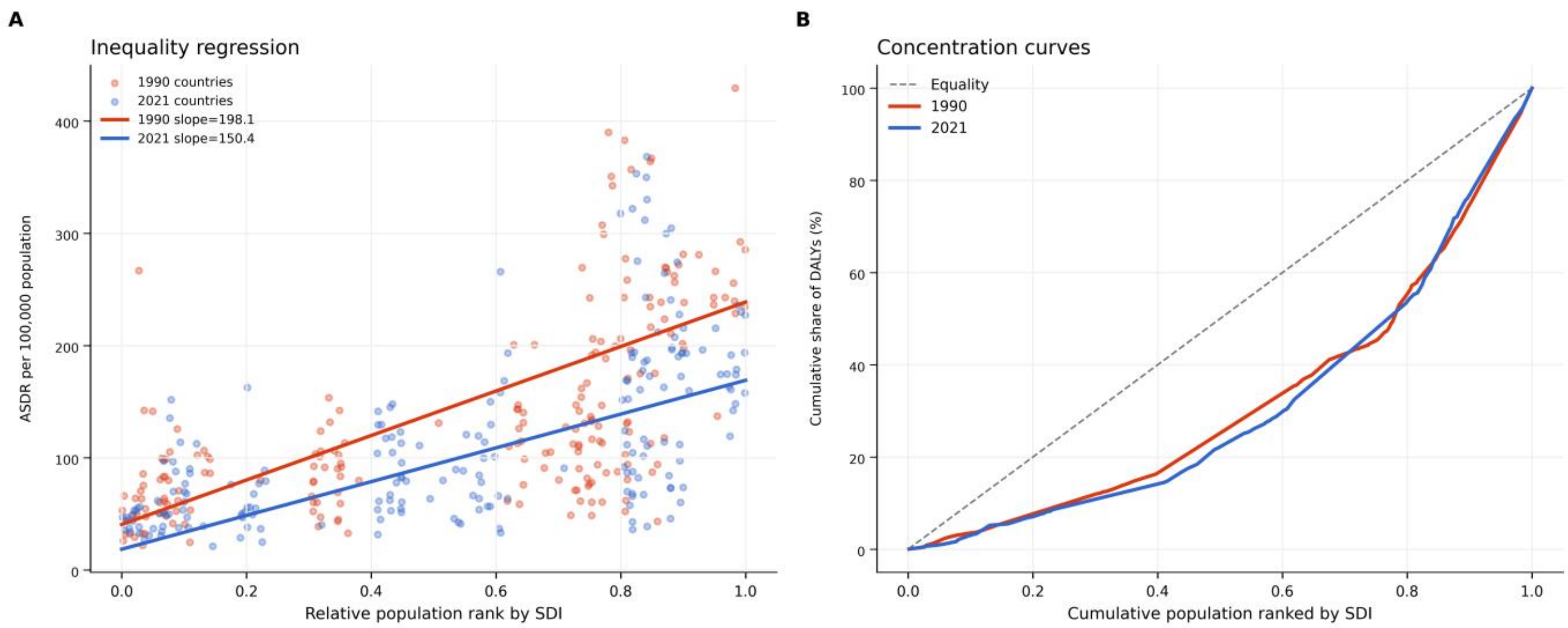

Note: Panel A shows regression-based inequality in ASDRs per 100,000 population across the SDI distribution. Panel B shows concentration curves for DALY counts ranked by SDI, with the y-axis expressed as cumulative share of DALYs (%). DALY = disability-adjusted life year; ASDR = age-standardized DALY rate; SDI = Socio-demographic Index; GBD = Global Burden of Disease.

Supplementary Figure S8. Global observed and projected age-standardized DALY rates for smoking-attributable musculoskeletal disorders in a secondary dataset analysis of GBD 2021 estimates, 1990-2050

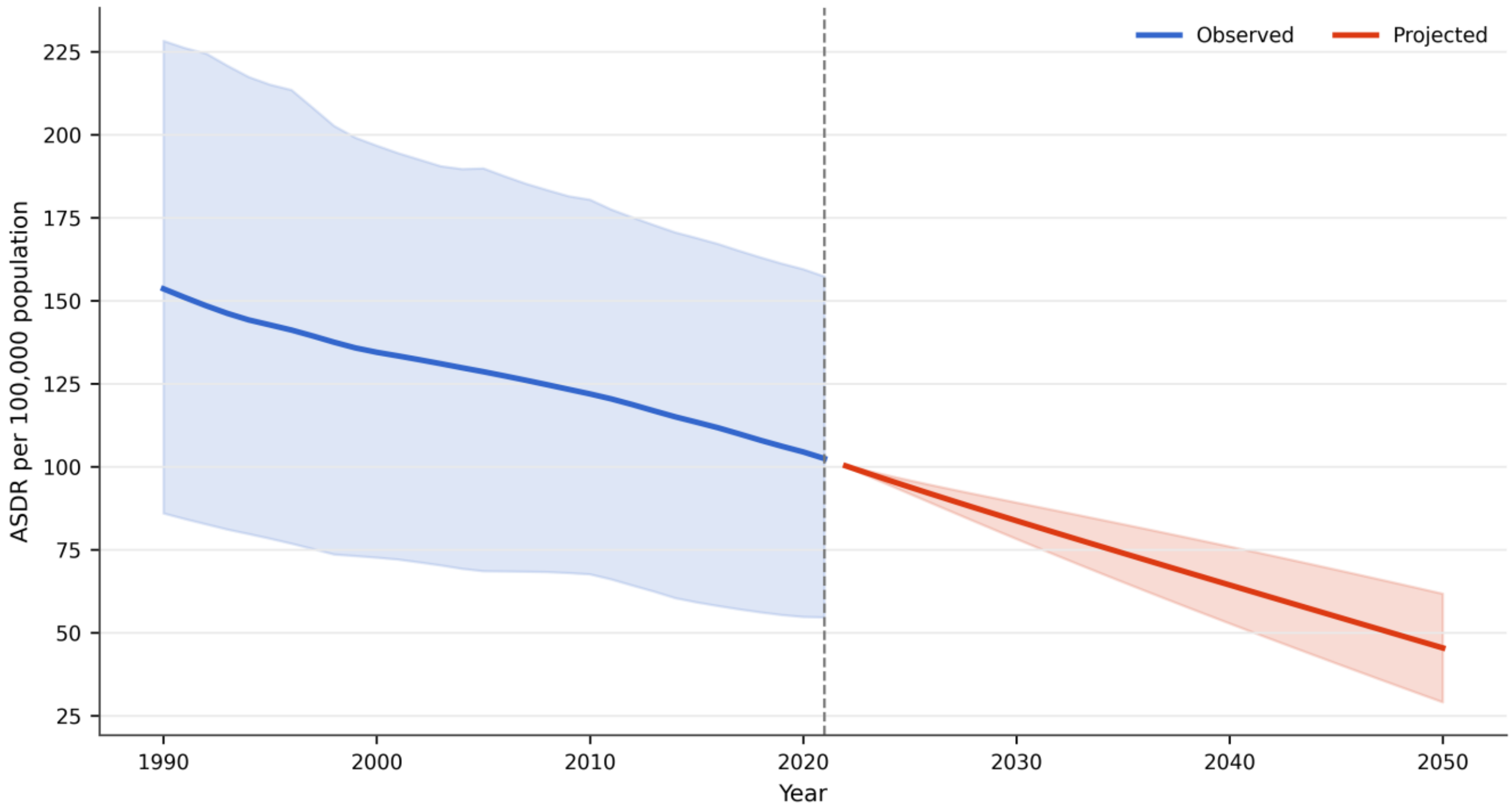

Note: The blue line shows observed ASDRs for 1990-2021, and the red line shows ARIMA-based exploratory projections for 2022-2050. Shaded areas indicate 95% uncertainty intervals. ASDR = age-standardized DALY rate per 100,000 population; DALY = disability-adjusted life year; ARIMA = autoregressive integrated moving average; GBD = Global Burden of Disease.

Supplementary Figure S9. Global observed and projected DALY counts for smoking-attributable musculoskeletal disorders in a secondary dataset analysis of GBD 2021 estimates, 1990-2050

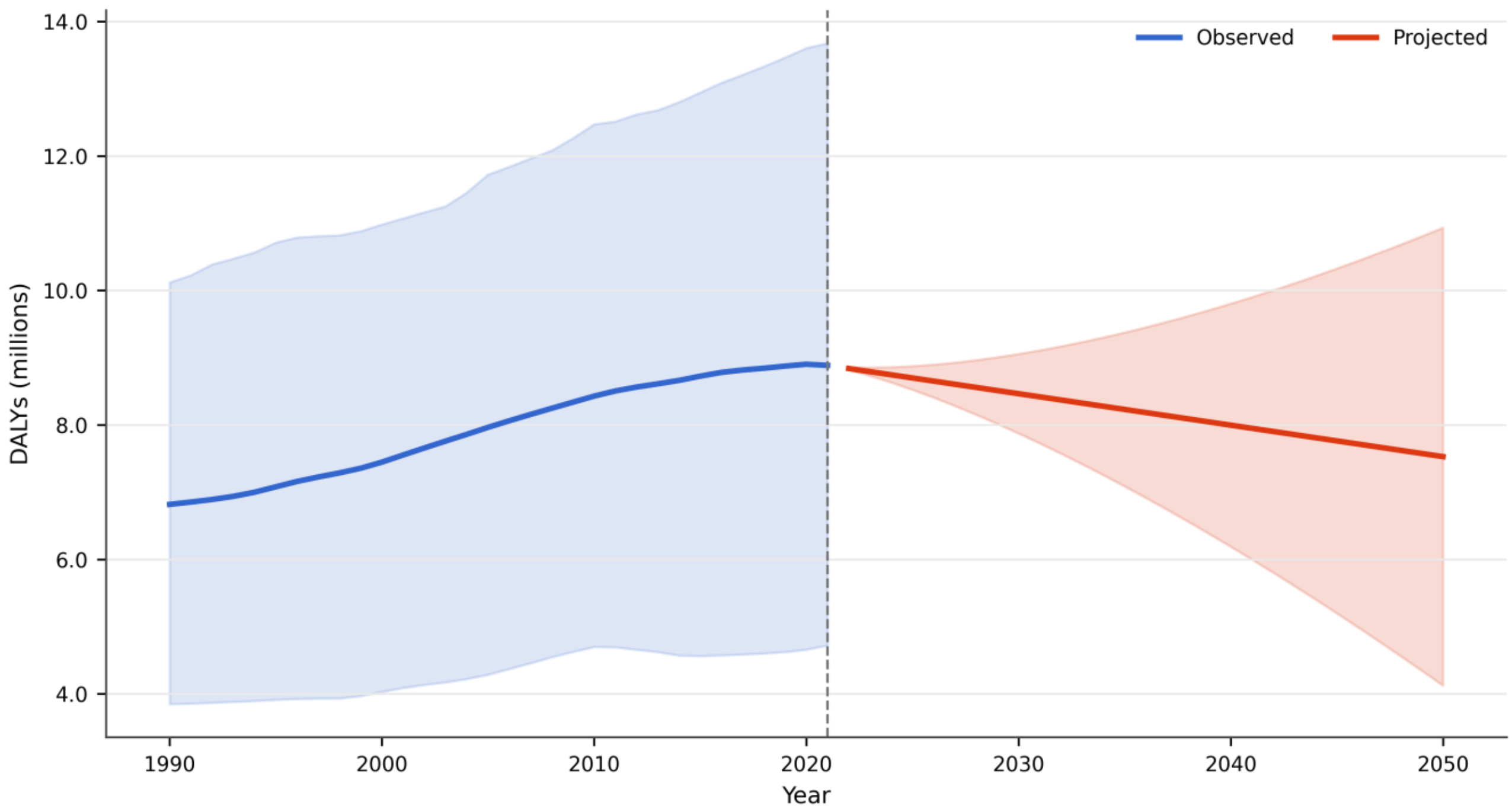

Note: The blue line shows observed DALY counts for 1990-2021, and the red line shows ARIMA-based exploratory projections for 2022-2050. Shaded areas indicate 95% uncertainty intervals. DALY = disability-adjusted life year; ARIMA = autoregressive integrated moving average; GBD = Global Burden of Disease.
